# circPSD3 is a promising inhibitor of uPA system to inhibit vascular invasion and metastasis in hepatocellular carcinoma

**DOI:** 10.1186/s12943-023-01882-z

**Published:** 2023-10-26

**Authors:** Liangliang Xu, Peng Wang, Li Li, Lian Li, Yang Huang, Yanfang Zhang, Xiaobo Zheng, Pengsheng Yi, Ming Zhang, Mingqing Xu

**Affiliations:** 1 Division of Liver Surgery, Department of General Surgery, West China Hospital, Sichuan University, No. 37, Guoxue Lane, Wuhou District, Chengdu, Sichuan Province 610041 China; 2 Institute of Clinical Pathology, West China Hospital of Sichuan University, Chengdu, 610041 China; 3 Center of Infectious Diseases, West China Hospital, Sichuan University, Chengdu, Sichuan Province 610041 China; 4 Department of Critical Care Medicine, West China Hospital, Sichuan University, Chengdu, Sichuan Province 610041 China; 5 Department of Hepato-biliary-pancrease, Affiliated Hospital of North Sichuan Medical College, Nanchong, Sichuan Province 637000 China; 6 Department of Hepatopancreatobiliary Surgery, Meishan City People’s Hospital, Meishan Hospital of West China Hospital, Sichuan University, Meishan, 620000 China

**Keywords:** Hepatocellular carcinoma, circPSD3, HDAC1, uPA system, Vascular invasion

## Abstract

**Background:**

Vascular invasion is a major route for intrahepatic and distant metastasis in hepatocellular carcinoma (HCC) and is a strong negative prognostic factor. Circular RNAs (circRNAs) play important roles in tumorigenesis and metastasis. However, the regulatory functions and underlying mechanisms of circRNAs in the development of vascular invasion in HCC are largely unknown.

**Methods:**

High throughput sequencing was used to screen dysregulated circRNAs in portal vein tumor thrombosis (PVTT) tissues. The biological functions of candidate circRNAs in the migration, vascular invasion, and metastasis of HCC cells were examined in vitro and in vivo. To explore the underlying mechanisms, RNA sequencing, MS2-tagged RNA affinity purification, mass spectrometry, and RNA immunoprecipitation assays were performed.

**Results:**

circRNA sequencing followed by quantitative real-time PCR (qRT-PCR) revealed that circRNA pleckstrin and Sect. 7 domain containing 3 (circPSD3) was significantly downregulated in PVTT tissues. Decreased circPSD3 expression in HCC tissues was associated with unfavourable characteristics and predicted poor prognosis in HCC. TAR DNA-binding protein 43 (TDP43) inhibited the biogenesis of circPSD3 by interacting with the downstream intron of pre-PSD3. circPSD3 inhibited the intrahepatic vascular invasion and metastasis of HCC cells in vitro and in vivo. Serpin family B member 2 (SERPINB2), an endogenous *bona fide* inhibitor of the urokinase-type plasminogen activator (uPA) system, is the downstream target of circPSD3. Mechanistically, circPSD3 interacts with histone deacetylase 1 (HDAC1) to sequester it in the cytoplasm, attenuating the inhibitory effect of HDAC1 on the transcription of SERPINB2. In vitro and in vivo studies demonstrated that circPSD3 is a promising inhibitor of the uPA system.

**Conclusions:**

circPSD3 is an essential regulator of vascular invasion and metastasis in HCC and may serve as a prognostic biomarker and therapeutic target.

**Supplementary Information:**

The online version contains supplementary material available at 10.1186/s12943-023-01882-z.

## Introduction

Hepatocellular carcinoma (HCC) is the most common histological subtype of primary liver cancer with high incidence and poor prognosis [1]. By the year 2025, liver cancer is projected to impact over 1 million individuals annually [2]. Hepatitis B virus (HBV) infection is the leading risk factor for the development of HCC, contributing to approximately 50% of cases [3]. Due to the achievement of sustained virological response (SVR) through the use of antiviral drugs, the risk associated with hepatitis C virus (HCV) infection has markedly declined [4]. Nevertheless, patients with cirrhosis remain at a heightened risk for the development of HCC even after HCV clearance. Additionally, non-alcoholic steatohepatitis (NASH), which is linked to metabolic syndrome or diabetes mellitus, is emerging as the rapidly growing cause of HCC, particularly in Western countries [5]. Metastasis is a defining characteristic of cancer [6]. HCC tends to invade the vascular system to form microvascular invasion (MVI) or portal vein tumor thrombosis (PVTT) [7]. Vascular invasion is a major route for intrahepatic and distant metastasis in HCC and is a strong negative prognostic factor [8]. PVTT is present in 10–60% of patients at the time of initial diagnosis of HCC [9]. Most treatment guidelines classify patients with PVTT as advanced stage with limited treatment options [2]. The median survival time of patients with PVTT treated with supportive care only is just 2–4 months [10]. Except for a small number of patients with simultaneously resectable HCC and PVTT who may benefit from surgery, the outcomes of non-surgical treatment for PVTT are unsatisfactory [11]. Hence, better understanding of the molecular mechanisms of vascular invasion is needed to develop more effective therapeutic approaches for PVTT.

Circular RNA (circRNA) is a new class of RNA that is generated by an alternative splicing method called back-splicing [12]. Due to the loop-like structure and because they lack poly(A) tails, circRNAs are resistant to RNase R degradation and are more stable than linear RNAs [13]. circRNAs exert their biological functions through various mechanisms, such as acting as microRNA (miRNA) sponges, interacting with proteins, and translating novel polypeptides [14]. circRNAs broadly exist in various eukaryotes and play important roles in organ development and disease progression, especially tumorigenesis and metastasis [15]. We previously showed that ciRS-7 is an independent predictor of MVI and circSETD3 inhibits the proliferation of HCC cells by sponging miR-421 [16, 17]. Hu et al. [18] identified circASAP1 as a key regulator of metastasis that may serve as a prognostic biomarker based on circRNA sequencing (circRNA-seq) of patients with HCC and postoperative pulmonary metastases. However, circRNAs that regulate vascular invasion in HCC are unknown.

Given that MVI is only observed under a microscope during pathological diagnosis, studies on MVI tissues are difficult [19]. Unlike MVI, PVTT can be detected by radiological examination and staged according to the site of thrombosis [20]. As patients with simultaneously resectable HCC and PVTT can derive a survival benefit from surgery [8], paired HCC and PVTT tissues harvested during surgery provide a valuable opportunity to study the mechanisms involved in the vascular invasion and metastasis of HCC. RNA sequencing (RNA-seq) has identified numerous dysregulated messenger RNAs (mRNAs) [21], miRNAs [22], and long non-coding RNAs (lncRNAs) [23] in PVTT, some of which have been shown to play an important role in HCC metastasis. In this study, using high throughput circRNA-seq, we showed that circRNA pleckstrin and Sect. 7 domain containing 3 (circPSD3) (derived from exons 13 and 14 of *PSD3*; circBase ID: hsa_circ_0136098) was significantly downregulated in PVTT tissues. Mechanistically, circPSD3 inhibited the migration and invasion of HCC cells in a urokinase-type plasminogen activator (uPA) system-dependent manner.

## Materials and methods

### HCC samples

In total, 159 HCC samples were obtained from the West China Biobanks, Department of Clinical Research Management, West China Hospital, Sichuan University. Nineteen patients had paired PVTT tissues and 48 patients had paired non-cancerous liver tissues. All patients underwent radical resection between 2016 and 2018. None of the patients received other preoperative anticancer treatments. The study was approved by the Biomedical Ethics Committee of West China Hospital (Ethic approval ID: 2022(1685)). Written informed consent was obtained from each patient.

### circRNA-seq

Five PVTT and matched HCC tissues were selected for RNase R-treated circRNA-seq (Novogene, Beijing, China). Briefly, 5 µg of high-quality RNA per sample was used as input material. Ribosomal and linear RNAs were removed before library preparation. circRNAs were identified using find_circ and CIRI. The R package “DESeq2” (version 2.15.13) was employed to screen differentially expressed circRNAs between PVTT and matched HCC tissues. circRNAs with |log2(fold-change)| >2.5 and FDR < 0.05 were considered to be differentially expressed.

### RNA-seq with unique molecular identifiers (UMIs)

To determine downstream targets and pathways of circPSD3, RNA-seq was performed using UMIs (Seqhealth Technology Co. Ltd., Wuhan, China). Briefly, 2 µg of total RNA extracted from HCC-LM9 cells stably transfected with circPSD3 overexpression or control lentivirus was used for stranded RNA-seq library preparation (Catalogue #DR08502; KC-Digital^™^ Stranded mRNA Library Prep Kit for Illumina, Seqhealth Technology Co. Ltd.), following the manufacturer’s instructions. The kit eliminates duplication bias in PCR and sequencing steps by using the UMIs of eight random bases to label the pre-amplified cDNA molecules. Library products corresponding to 200–500 bps were enriched, quantified, and sequenced on a NovaSeq 6000 sequencer (PE150 model) (Illumina, San Diego, CA, USA). Raw sequencing data were filtered using Trimmomatic (version 0.36). Clean reads were further treated with in-house scripts to eliminate duplication bias introduced in library preparation and sequencing. TopHat2 (version 2.0.13) was used to align de-duplicated reads to the reference genome (GRCh38/hg38). The R package “DESeq2” (version 2.15.13) was used to identify differentially expressed genes between groups. Genes with |log2(fold-change)| >1 and FDR < 0.05 were considered to be differentially expressed. The molecular functions and pathways of dysregulated genes were analysed in KOBAS 2.0.

### Cell culture and transfection

Human HCC cell lines (HCC-LM9 and SK-Hep-1) cells were maintained in Dulbecco’s modified Eagle medium/high-glucose medium (HyClone, Logan, UT, USA) supplemented with 10% foetal bovine serum (FBS) (PAN-Biotek, Aidenbach, Bavaria) and antibiotics (1% penicillin/streptomycin; HyClone) in a humidified incubator with 5% CO_2_ at 37ºC.

Small interfering RNAs (siRNAs) targeting circPSD3, TAR DNA-binding protein 43 (TDP43), serpin family B member 2 (SERPINB2), and histone deacetylase 1 (HDAC1) were designed and synthesised by RiboBio (Guangzhou, China). The siRNA sequences are listed in Table S1 (Additional file 1). siRNAs were transfected using GeneMute (SignaGen Laboratories, Rockville, MD, USA), according to the manufacturer’s instructions. The lentiviruses used to express circPSD3 and uPA receptor (uPAR) were constructed by GeneChem (Shanghai, China) and infected following the manufacturer’s protocol.

### Quantitative real-time (qRT)-PCR

Genomic DNA (gDNA) was isolated using the PureLink™ Genomic DNA Mini Kit (Thermo Fisher Scientific, Waltham, MA, USA), according to the manufacturer’s instructions. Total RNA was extracted using TRIzol (Invitrogen Life Technologies Inc., Germany). Reverse transcription was performed using HiScript III RT SuperMix for qPCR (+ gDNA wiper) Kit (Vazyme Biotech Co. Ltd., Nanjing, China). Nuclear and cytoplasmic fractions were isolated using the PARIS™ Kit (Thermo Fisher Scientific). qRT-PCR was performed in triplicate using 2× ChamQ Universal SYBR qPCR Master Mix (Vazyme Biotech Co. Ltd.) and the CFX Connect Real-Time System (Bio-Rad, Hercules, CA, USA). The primers used in this study are listed in Table S2 (Additional file 1).

### Fluorescence in situ hybridisation (FISH)

Cy3-labelled probes were synthesised by RiboBio. FISH was performed using the FISH Kit (RiboBio), according to the manufacturer’s instructions. Images were captured using the A1RþMP Confocal Laser Microscope System (Nikon, Tokyo, Japan). U6 and 18 S ribosomal RNAs (rRNAs) were used as positive controls in the nucleus and cytoplasm, respectively.

### Actinomycin D and RNase R treatment assays

circRNA stability was assessed using actinomycin D and RNase R treatment assays. For actinomycin D assay, HCC cells were cultured in medium containing 2 mg/mL actinomycin D. Total RNA was isolated at the indicated time points and subjected to qRT-PCR. For RNase R treatment, 3 µg of total RNA was treated with 10 U RNase R (20 U/µL; Epicenter, Madison, WI, USA) at 37 °C for 45 min, followed by 70 °C for 10 min to deactivate RNase R. Linear and circular RNA degradation was determined by PCR followed by agarose gel electrophoresis.

### Western blotting and immunohistochemistry

Western blotting was performed as described previously [17, 24]. The following primary antibodies were used: anti-SERPINB2 (1:1,000) (Proteintech, Wuhan, China), anti-uPAR (1:1,000) (Abcam, Cambridge, UK), anti-uPA (1:1,000) (Abcam), anti-HDAC1 (1:1,000) (Abclonal, Wuhan, China), anti-AGO2 (1:1,000) (Abclonal), anti-GST (1:1,000) (Abcam), anti-FLAG (1:1,000) (Abclonal), and anti-TDP43 (1:1,000) (Proteintech). Immunohistochemistry was performed using antibodies against TDP43 (1:200) (Proteintech), SERPINB2 (1:200) (Proteintech), and uPAR (1:200) (Abcam), as described previously [17, 24].

### Immunofluorescence staining

Cells were seeded into a 24-well plate with a coverslip at the bottom of each well. After incubation for 24 h at 37 °C, the cells were fixed with 4% paraformaldehyde for 30 min, permeabilised with 0.2% Triton X-100 for 10 min, and blocked with 3% bovine serum albumin for 1 h. Cells were incubated with specific antibodies at 4 °C overnight and with fluorescence-conjugated secondary antibodies at 37 °C for 1 h. Nuclei were stained with 4’6-diamidino-2-phenylindole (DAPI) for 10 min. After sealing, images were acquired using a confocal laser scanning microscope (Nikon).

### Cell viability assay

Cell viability was assessed using the Cell Counting Kit-8 (CCK-8) and colony formation assays. For CCK-8 assay, HCC cells were suspended in 100 µL of medium and seeded into 96-well plates at a density of 2,000 cells/well. After incubation with 10 µL of CCK-8 solution for 1.5 h, absorbance at 450 nm was measured at indicated time points using the Eon^™^ Microplate Reader (BioTek, Whiting, VT, USA). For colony formation assay, 1,000 cells were seeded into each well of a 6-well plate. After incubation for 14 days, the colonies were fixed with 4% paraformaldehyde (Solarbio, Beijing, China) and stained with 0.05% crystal violet (Beyotime Biotechnology, Nantong, China). Colonies were photographed and counted. All experiments were performed in triplicate.

### Wound healing assay

Confluent monolayer cells in 6-well plates were wounded using a 200 µL pipette tip. After washing with PBS twice, cells were cultured in medium containing 3% FBS. Images were acquired using an inverted microscope (Carl Zeiss, Jena, Germany) at 0 and 48 h after wounding. At least three separate fields were photographed. The relative healed area was calculated using ImageJ (National Institutes of Health, Bethesda, MD, USA) and normalised to 0 h control.

### Transwell migration and Matrigel invasion assays

For Transwell migration assay, 3 × 10^4^ HCC cells were suspended in 300 µL of serum-free medium and seeded into the upper chamber (pore size, 8 μm) (Millipore, Billerica, MA, USA). The bottom chamber contained 600 µL of medium containing 10% FBS as a chemoattractant. After 24 h, cells on the lower surface of the upper chamber were fixed with 4% paraformaldehyde, stained with 0.05% crystal violet, and imaged at 100× magnification. At least three random fields were photographed. Migrated cell numbers were counted using Image J. A similar protocol was performed for Matrigel invasion assay, except 30 µL of diluted Matrigel (BD Bioscience, Bedford, MA, USA) was added to the upper chamber before cell seeding.

### 3D spheroid-based Matrigel invasion assay

Complete growth medium containing 1 × 10^4^ SK-Hep-1 cells (200 µL) was seeded into ultralow attachment 96-well round-bottom plates (Corning, USA) for 3 days, as described previously [25–27]. Images were acquired when tumor spheroids were formed (Day 0). Then, 100 µL of plating medium was removed and replaced with the same volume of Matrigel. After Matrigel solidification, 100 µL of complete growth medium was added to each well. After 3 days, images were photographed and analysed using the Celigo^™^ cytometer (Nexcelom Bioscience, Lawrence, MA, USA).

### RNA immunoprecipitation (RIP) assay

RIP assay was performed using the Magna RIP™ RNA-Binding Protein Immunoprecipitation Kit (Millipore), according to the manufacturer’s instructions. Briefly, magnetic beads were sequentially incubated with primary antibodies and prepared cell lysates. Enriched RNA was isolated using TRIzol reagent and quantified by qRT-PCR. Immunoglobulin G antibody served as the negative control.

### MS2-tagged RNA affinity purification (TRAP) assay

A 2× MS2 stem–loop sequence was inserted at the back-splicing site of circPSD3 (Biosense, Guangzhou, China). The MS2-circPSD3 expression plasmid or negative control plasmid was co-transfected with the GST-MS2 expression plasmid into HCC cells. The cell lysates were incubated with anti-GST-coated magnetic beads (Beyotime Biotechnology) at 4 °C overnight. Enriched RNAs and proteins were collected. RNA was quantified by qRT-PCR. Proteins were quantified by liquid chromatography–tandem mass spectrometry (LC-MS/MS) (OEBiotech, Shanghai, China) and validated by western blotting.

### Dual-luciferase reporter assay

A dual-luciferase reporter plasmid was constructed as a vector containing Renilla luciferase (Rluc) and firefly luciferase (Luc) (VectorBuilder, Guangzhou, China). The predicted internal ribosome entry site (IRES) of circPSD3 or mutant sequences was subcloned into the promoter region of Luc. The plasmid was transfected into HEK-293T cells using Lipo6000^™^ Transfection Reagent (Beyotime Biotechnology), following the manufacturer’s instructions. After incubation for 48 h, Rluc and Luc activity was determined using the Duo-Lite Luciferase Assay System (Vazyme Biotech Co. Ltd.). Results are presented as the ratio of Luc to Rluc.

### Animal metastasis models

Male BALB/c nude mice (5–6 weeks old) were purchased from HFK Bioscience (Beijing, China) and maintained under specific pathogen-free conditions. All animal experiments were approved by the Animal Care Committee of Sichuan University (Ethic approval ID: 20,220,224,052). To establish a lung metastasis model, HCC cells were injected into the tail vein of nude mice. Five weeks later, the mice were anaesthetised. Resected lung specimens were stained with haematoxylin and eosin (H&E). To establish an intrahepatic metastasis model, 2 × 10^6^ stably transfected cells were injected into the liver of nude mice. Six weeks later, liver lobes containing tumors were harvested and subjected to H&E staining. Serial sectioning of liver tissues was performed to confirm vascular invasion. Pathological images were acquired using a Whole Slide Image Scanner (Unic Technologies Inc., Beijing, China). The number of metastatic nodules and vascular invasion were determined by two independent investigators. At least five mice were included in each group. All animal experiments were conducted in accordance with the Guide for the Care and Use of Laboratory Animals.

### Statistical analysis

Statistical analyses were conducted using SPSS (version 21.0) (IBM Corp., Armonk, NY, USA), GraphPad Prism (version 8.0) (GraphPad Software, La Jolla, CA, USA), and R (version 3.5.0) (The R Foundation, Vienna, Austria; http://www.r-project.org/). Continuous variables were expressed as mean ± SD and compared using Student’s *t*-test. Categorical variables were expressed as numbers and percentages and compared using the chi-square test or Fisher’s exact test, as appropriate. Correlations were determined using Pearson’s correlation coefficients. The optimal cut-off value for circPSD3 expression in HCC tissues was determined using X-tile software. Survival curves were plotted using the Kaplan–Meier method and compared using the log-rank test. All statistical tests were two-tailed. A p-value < 0.05 was considered statistically significant.

## Results

### CircPSD3 is a potential regulator of PVTT formation

**Fig. 1:**
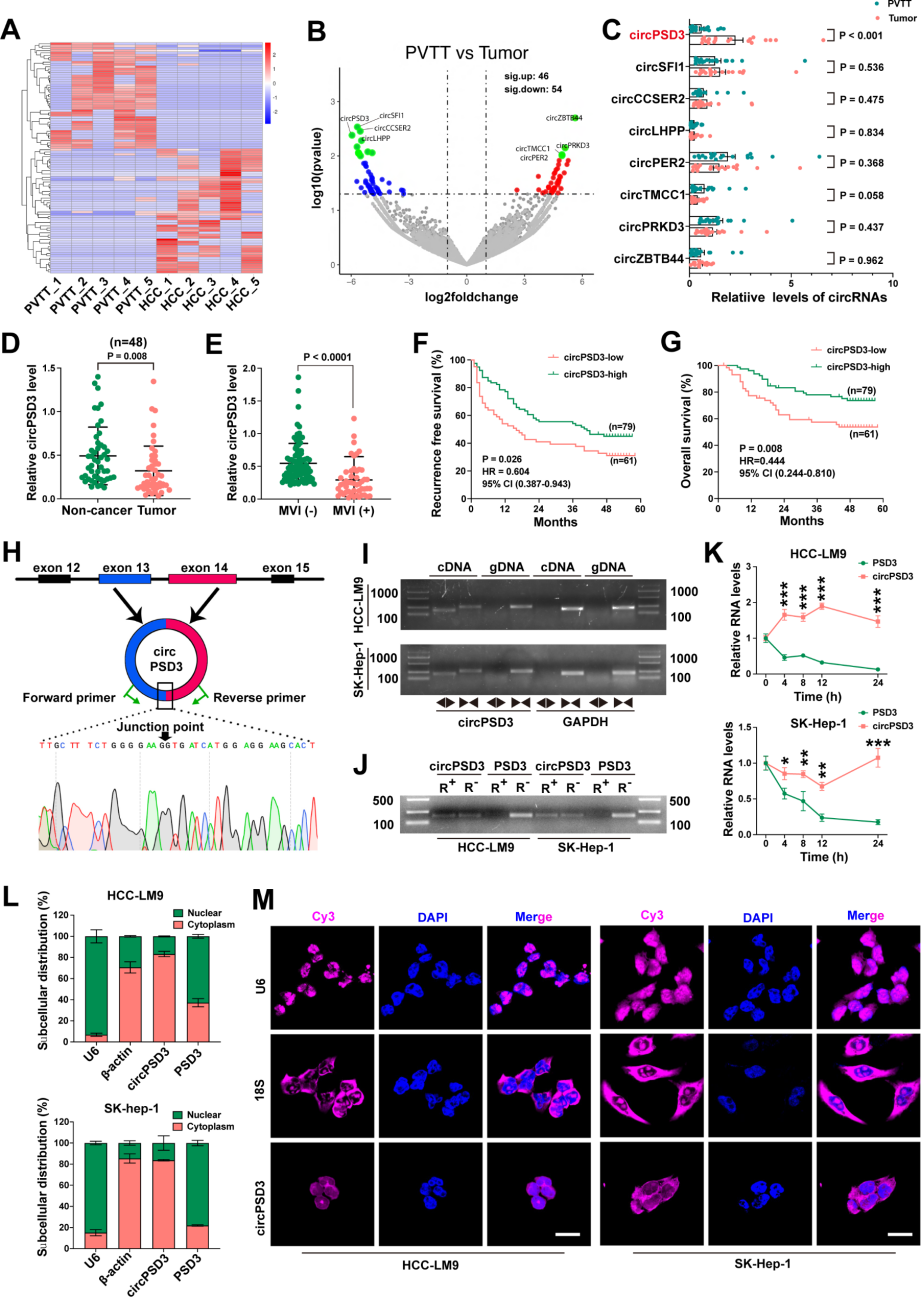
circPSD3 is downregulated in PVTT tissues and is associated with the prognosis of patients with HCC. (**A**) Clustered heatmap of differentially expressed circRNAs in five paired PVTT and HCC tissues. Rows represent circRNAs while columns represent tissues. (**B**) Volcano plot of dysregulated circRNAs in paired PVTT and HCC tissues. (**C**) qRT-PCR validation of the top four up and downregulated circRNAs in 19 paired PVTT tissues. (**D**) Relative expression of circPSD3 in 48 paired HCC and non-cancerous tissues. (**E**) Differences in circPSD3 expression between HCC tissues with and without MVI. (**F** and **G**) Kaplan–Meier curves showing the correlation between circPSD3 expression and recurrence-free survival and overall survival. Survival curves were compared using the log-rank test. (**H**) Schematic showing that exons 13 and 14 of human *PSD3* circularise to produce circPSD3. The back-splice junction site was confirmed by Sanger sequencing. (**I**) The circular structure of circPSD3 was confirmed in HCC cell lines using convergent and divergent primers. *GAPDH* served as the reference gene. (**J**) Circular and linear isoform stability was assessed using RNase R treatment. (**K**) Relative RNA levels of circPSD3 and linear PSD3 in HCC cell lines treated with actinomycin D. (**L**) The subcellular localisation of circPSD3 in HCC cell lines using nuclear/cytoplasmic RNA fractionation. U6 and β-actin served as the nuclear and cytoplasmic controls, respectively. (**M**) FISH visualisation of the subcellular localization of circPSD3 in HCC cell lines. U6 and 18 S ribosomal RNA served as the nuclear and cytoplasmic controls, respectively. Scale bar = 20 μm. Data are shown as the mean ± SD. Statistical analyses were performed using unpaired Student’s t-tests (*p < 0.05; **p < 0.01; ***p < 0.001). circPSD3, circRNA pleckstrin and Sect. 7 domain containing 3; FISH, fluorescence in situ hybridisation; HCC, hepatocellular carcinoma; MVI, microvascular invasion; PVTT, portal vein tumor thrombosis; qRT-PCR, quantitative real-time PCR

To screen for circRNAs involved in vascular invasion in HCC, we performed circRNA-seq on five paired PVTT and HCC tissues. A total of 13,238 circRNAs (distributed across all chromosomes) were detected (Additional file 1: Figure S1A; Additional file 2: Table S3). Among them, 8,523 were annotated in circBase. The remaining 4,715 were considered novel. Most circRNAs ranged from 200 to 600 bp in length and were derived from exons (Additional file 1: Figure S1B, C). Based on the criteria of |log2(fold-change)| >2.5 and p < 0.05, 100 differentially expressed circRNAs were identified, including 46 that were significantly upregulated and 54 that were significantly downregulated in PVTT tissues (Fig. 1A, B). However, None of the differentially expressed circRNAs had an adjusted p-value < 0.05, indicating high heterogeneity among PVTT tissues (Additional file 3: Table S4), consistent with lncRNA-seq [23].

To further ascertain circRNAs associated with vascular invasion, eight of the most significantly dysregulated circRNAs (four upregulated and four downregulated) were selected and validated by qRT-PCR in 19 paired PVTT and HCC tissues (Fig. 1C). The melt curves of qRT-PCR and agarose gel electrophoresis of PCR products demonstrated the specificity of divergent primers against these circRNAs (Additional file 1: Fig. S1D and E). Sanger sequencing further confirmed that these primers amplified the target circRNAs (Additional file 1: Fig. S1F). Our results show that only circPSD3 was significantly downregulated in PVTT tissues compared with matched HCC tissues (Fig. 1C). circPSD3 was also the most significantly downregulated circRNA in circRNA-seq.

Next, we performed qRT-PCR to measure the expression levels of circPSD3 in another cohort comprising 140 human HCC tissues, 48 of which had paired non-cancerous tissues. As expected, circPSD3 was significantly downregulated in HCC tissues compared with non-cancerous tissues (Fig. 1D). The expression levels of circPSD3 in the MVI group were significantly lower than those in the non-MVI group (Fig. 1E), indicating that circPSD3 may be associated with vascular invasion. The optimal cut-off value of circPSD3 in HCC tissues was 0.465 for both overall survival and recurrence. Using this cut-off value, patients were divided into a low circPSD3 group (n = 61) and a high circPSD3 group (n = 79). Kaplan–Meier curves showed that decreased circPSD3 expression was associated with significantly poorer recurrence-free survival and overall survival in patients with HCC (Fig. 1F, G). The expression levels of circPSD3 in HCC tissues were associated with various clinicopathological characteristics, including elevated alpha-fetoprotein levels, multiple tumors, and the presence of MVI (Additional file 1: Table S5). Therefore, circPSD3 was selected as a candidate circRNA for the regulation of vascular invasion and metastasis in HCC and investigated in depth.

As shown in Fig. 1H, circPSD3 was back-spliced by exons 13 and 14 of *PSD3* located on chromosome 8 with a length of 1,504 nucleotides. To confirm the circular property of circPSD3, a series of experiments were performed. First, the back-splice junction site of circPSD3 was confirmed by Sanger sequencing in HCC cells (Fig. 1H). Second, circPSD3 could only be amplified from cDNA and not from gDNA using divergent primers, whereas the corresponding linear transcript could be amplified from both cDNA and gDNA using convergent primers, with GAPDH as the reference gene, indicating that circPSD3 was generated by head-to-tail splicing rather than by trans-splicing or genomic rearrangement (Fig. 1I). Third, actinomycin D and RNase R treatment assays demonstrated that circPSD3 was more stable than linear PSD3 (Fig. 1J, K). Finally, nuclear and cytoplasmic RNA separation and FISH assays showed that circPSD3 was preferentially located in the cytoplasm of HCC cells (Fig. 1L, M).

### TDP43 inhibits the biogenesis of circPSD3 in HCC

**Fig. 2:**
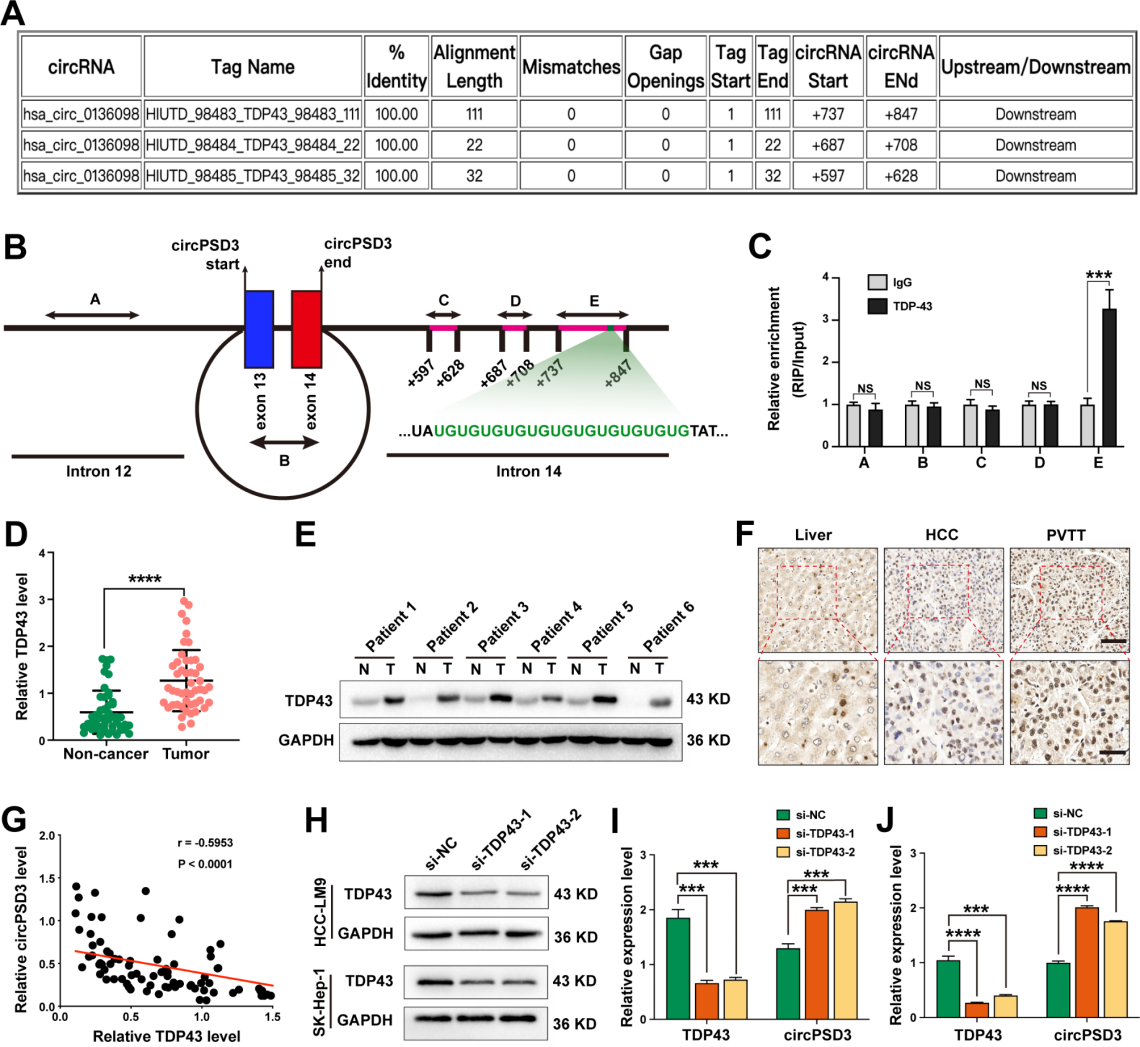
TDP43 inhibits the biogenesis of circPSD3. (**A**) Putative TDP43 binding sites in *PSD3* pre-mRNA were predicted using the online CircInteractome database. (**B**) Schematic showing the positions of qRT-PCR primers (A–E) on *PSD3* pre-mRNA. A (UG)_11_ repeat was identified at position E. (**C**) The direct binding of TDP43 to the *PSD3* pre-mRNA was confirmed using a RIP assay. (**D**) The relative mRNA levels of *TDP43* in 48 paired HCC and non-cancerous tissues were measured by qRT-PCR. (**E**) The protein levels of TDP43 in six paired HCC and non-cancerous tissues were measured using western blotting. (**F**) Immunohistochemical staining revealing the protein levels of TDP43 in a patient with HCC and PVTT. Scale bar, upper = 50 μm; lower = 20 μm. (**G**) Pearson correlation analysis showing a negative correlation between *TDP43* and circPSD3 in 48 HCC tissues. (**H**) Western blotting verification of TDP43 knockdown in HCC cell lines transfected with the indicated siRNAs. (**I** and **J**) Relative levels of circPSD3 in HCC-LM9 and SK-Hep-1 cells transfected with TDP43 siRNAs. Data are shown as the mean ± SD. Statistical analyses were performed using unpaired Student’s t-tests (NS, no statistical significance; ***p < 0.001; ****p < 0.0001). circPSD3, circRNA pleckstrin and Sect. 7 domain containing 3; HCC, hepatocellular carcinoma; mRNA, messenger RNA; PVTT, portal vein tumor thrombosis; qRT-PCR, quantitative real-time PCR; RIP, RNA immunoprecipitation; siRNA, small interfering RNA; TDP43, TAR DNA-binding protein 43;N, non-tumorous tissues; T, tumor tissues

Many RNA-binding proteins regulate the biogenesis of circRNAs by adhering to up or downstream introns flanking the back-splicing sites [28–30]. Using the online CircInteractome tool (https://circinteractome.nia.nih.gov/rna_ binding_protein.html), we found three putative binding sites of TDP43 in the downstream intron of circPSD3 pre-mRNA (Fig. 2A). TDP43 mediates several aspects of RNA metabolism, including RNA transcription, alternative splicing, and mRNA stabilisation, by interacting with UG repeats [31]. Herein, a (UG)_11_ repeat was identified in the third putative binding site of circPSD3 (Fig. 2B). To confirm the interaction between TDP43 and circPSD3 pre-mRNA, five primer pairs (A–E) were designed to target intron 12, the main body of circPSD3, and three TDP43-binding sites in intron 14, respectively (Fig. 2B). Next, a RIP assay was performed using an anti-TDP43 antibody. As expected, TDP43 only enriched sequences targeted by primer E (Fig. 2C), indicating that TDP43 formed a protein–RNA complex via the putative binding sites in circPSD3 pre-mRNA. Studies have shown that TDP43 is upregulated in HCC tissues and facilitates the degradation of HCC [32–34]. In this study, the mRNA level of *TDP43* was measured in 48 paired HCC samples. We found that circPSD3 was significantly upregulated in HCC tissues compared with non-cancerous tissues (Fig. 2D). This result was strengthened by western blotting in six paired HCC tissues (Fig. 2E). In a patient with HCC and PVTT, immunohistochemical staining showed that the density of TDP43 increased from non-cancerous to HCC and to PVTT tissues (Fig. 2F), demonstrating the stimulatory effect of TDP43 on vascular invasion in HCC. Pearson correlation analysis revealed that the expression of *TDP43* in HCC tissues was negatively correlated with circPSD3 (Fig. 2G). TDP43 depletion significantly increased circPSD3 expression in HCC cells (Fig. 2H–J). Taken together, these results suggest that TDP43 suppresses circPSD3 biogenesis and that circPSD3 may mediate the regulatory effect of TDP43 on the invasion and metastasis of HCC cells.

### circPSD3 inhibits the migration and invasion of HCC cells in vitro

**Fig. 3:**
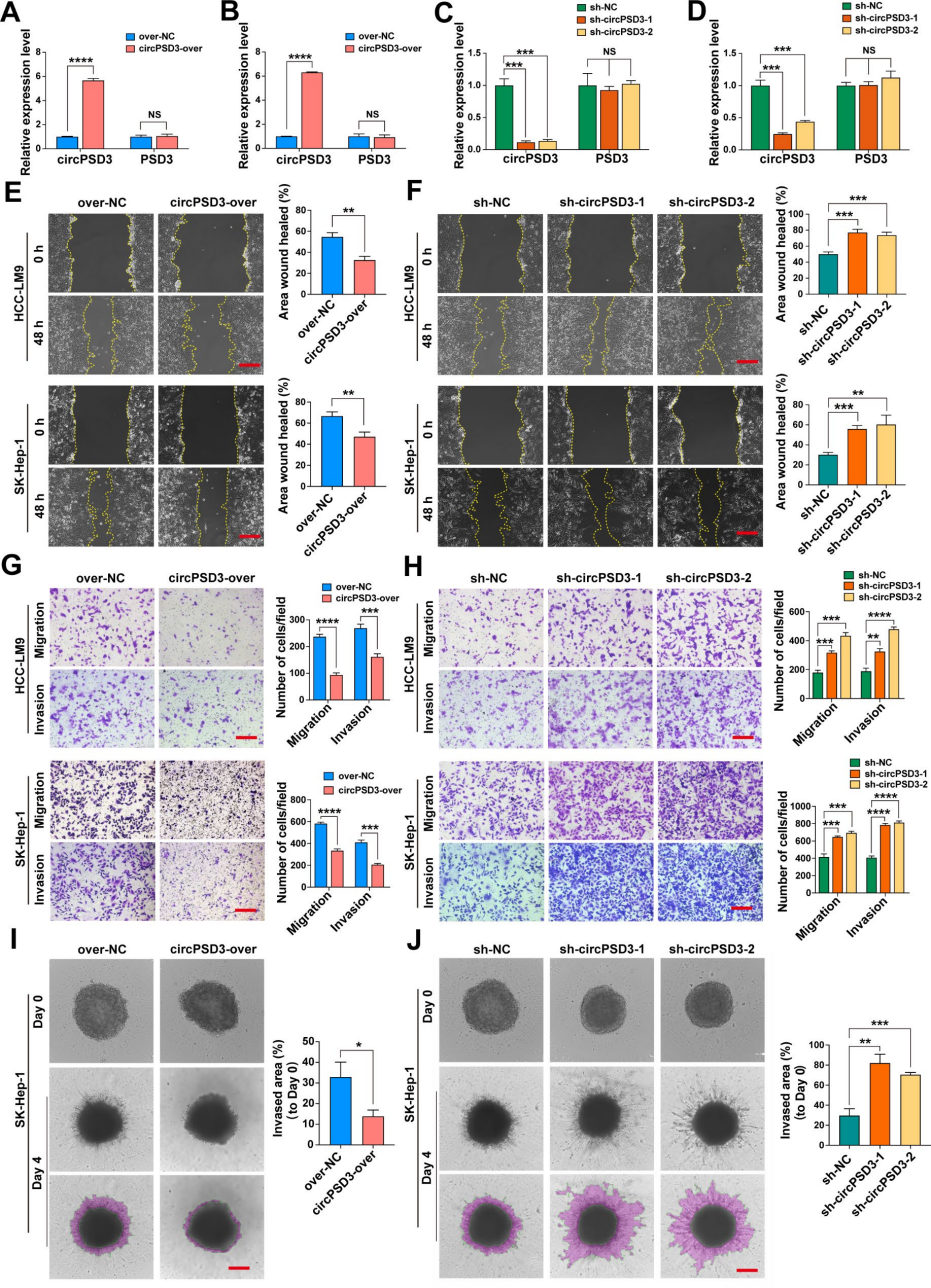
circPSD3 inhibits the migration and invasion of HCC cells in vitro. (**A** and **B**) The relative expression levels of circPSD3 and linear PSD3 in HCC-LM9 and SK-Hep-1 cells stably transfected with circPSD3 overexpression or control lentivirus were measured by qRT-PCR. (**C** and **D**) The relative expression levels of circPSD3 and linear *PSD3* in HCC-LM9 and SK-Hep-1 cells stably transfected with circPSD3 shRNA (sh-circPSD3-1/2) or scramble shRNA (sh-NC) lentivirus was measured by qRT-PCR. (**E** and **F**) Representative images and quantification of the wound healing assays in circPSD3-overexpressing, circPSD3 knockdown, and control HCC cells. scale bar = 100 μm. (**G** and **H**) Representative images and quantification of the Transwell migration and invasion assays in circPSD3-overexpressing, circPSD3 knockdown, and control HCC cells. scale bar = 100 μm. (**I** and **J**) Representative images and quantification of the 3D spheroid-based invasion assay in circPSD3-overexpressing and control HCC cells. scale bar = 100 μm. Data are shown as the mean ± SD. Statistical analyses were performed using unpaired Student’s t-tests (NS, no statistical significance; *p < 0.05; **p < 0.01; ***p < 0.001; ****p < 0.0001). circPSD3, circRNA pleckstrin and Sect. 7 domain containing 3; HCC, hepatocellular carcinoma; qRT-PCR, quantitative real-time PCR

To explore the effect of circPSD3 on HCC progression, the expression of circPSD3 was artificially changed using an overexpression lentivirus and two shRNA lentiviruses targeting the back-splicing site of circPSD3. After transfection, > 90% of cells expressed green fluorescent protein (Additional file 1: Fig. S2). qRT-PCR showed that circPSD3 was successfully overexpressed or knocked down in HCC cells with no effect on linear *PSD3* (Fig. 3A–D). HCC cell migration was significantly reduced in response to circPSD3 overexpression, and was significantly enhanced in response to circPSD3 knockdown, in the wound healing assays (Fig. 3E, F). HCC cell migration and invasion were diminished by circPSD3 overexpression, and enhanced by circPSD3 knockdown, in the Transwell assays (Fig. 3G, H). Moreover, a 3D spheroid-based invasion assay was performed to mimic HCC cell invasion in vivo. Stable overexpression of circPSD3 significantly reduced the invasion area and the number of protrusions, whereas circPSD3 depletion significantly increased the invasion area and the number of protrusions in SK-Hep-1 cells (Fig. 3I, J). Nevertheless, CCK-8 and colony formation assays showed that circPSD3 had no effect on the proliferation of HCC cells (Additional file 1: Fig. S3A–E). Taken together, these results suggest an inhibitory effect of circPSD3 on HCC cell migration and invasion, but not growth.

### circPSD3 suppresses distant metastasis, intrahepatic metastasis, and vascular invasion of HCC cells

**Fig. 4:**
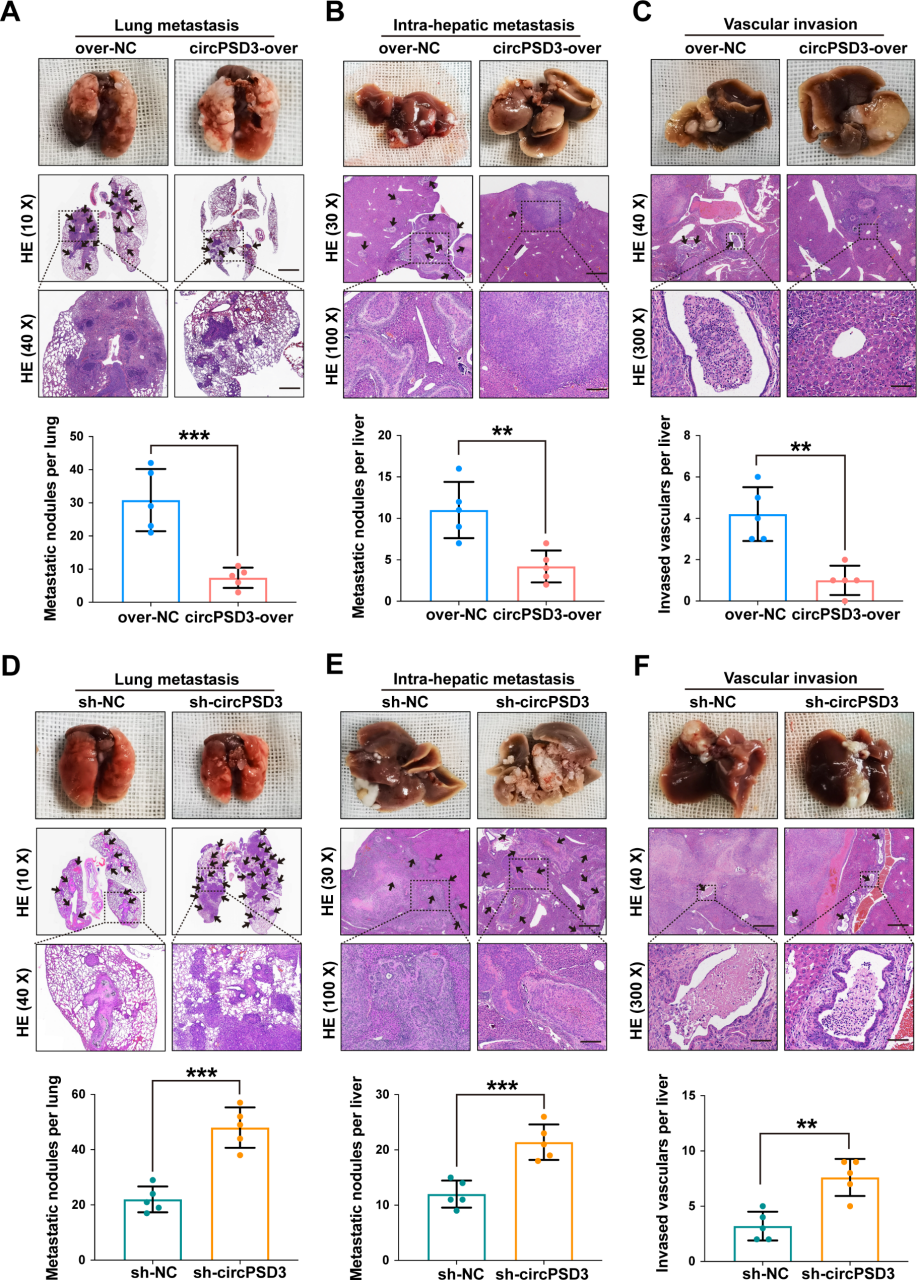
circPSD3 promotes the metastasis and vascular invasion of HCC cells in vivo. (**A**) Representative images and quantification of H&E staining of lung metastatic nodules of nude mice injected with circPSD3-overexpressing or control HCC-LM9 cells via the tail vein. Scale bar, upper = 1000 μm; lower = 400 μm. (**B**) Representative images and quantification of H&E staining of intrahepatic metastatic nodules in the circPSD3 overexpression and control groups. Scale bar, upper = 300 μm; lower = 100 μm. (**C**) H&E-stained serial liver sections showing intrahepatic vascular invasion in the circPSD3 overexpression and control groups. Scale bar, upper = 400 μm; lower = 30 μm. (**D–F**) Representative images and quantification of H&E staining of lung metastatic nodules, intrahepatic metastatic nodules, and intrahepatic vascular invasion in the circPSD3 knockdown and control groups. Each group contained 5 mice. Data are shown as the mean ± SD. Statistical analyses were performed using unpaired Student’s t-tests (**p < 0.01; ***p < 0.001). circPSD3, circRNA pleckstrin and Sect. 7 domain containing 3; HCC, hepatocellular carcinoma; H&E, haematoxylin and eosin

To strengthen the findings of the in vitro studies, pulmonary metastasis and liver orthotopic implantation models were constructed. Whole-slide scans of H&E-stained sections showed that overexpression of circPSD3 significantly reduced the number of lung metastatic lesions (Fig. 4A). Overexpression of circPSD3 also significantly reduced intrahepatic metastasis (Fig. 4B). To evaluate the effect of circPSD3 on intrahepatic vascular invasion, serial sectioning of liver tissues and H&E staining were performed. The results showed that overexpression of circPSD3 significantly reduced the vascular invasion ability of HCC cells (Fig. 4C). Conversely, circPSD3 knockdown significantly increased lung metastasis, intrahepatic metastasis, and intrahepatic vascular invasion (Fig. 4D–F). Taken together, these results suggest that circPSD3 plays an important role in inhibiting the vascular invasion and metastasis of HCC cells and is a promising therapeutic target.

### circPSD3 regulates the uPA system

**Fig. 5:**
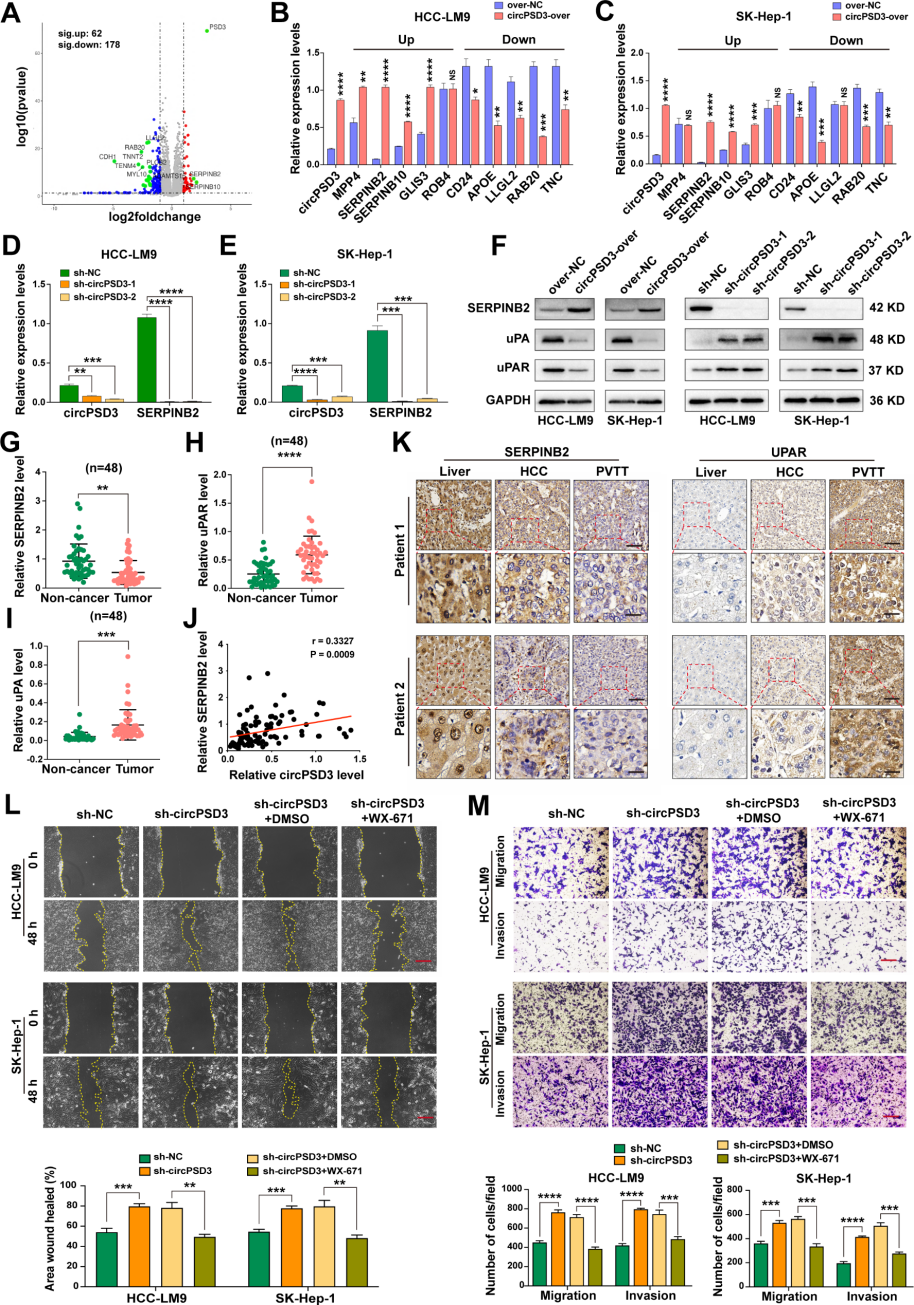
circPSD3 regulates the uPA system. (**A**) Volcano plot of differentially expressed genes between the circPSD3-overexpression and control groups. (**B** and **C**) qRT-PCR validation of the top five up and downregulated genes in HCC-LM9 and SK-Hep-1 cells. (**D** and **E**) circPSD3 knockdown of HCC-LM9 and SK-Hep-1 cells demonstrating the regulatory relationship between circPSD3 and SERPINB2. (**F**) Western blotting analysis of uPA system protein expression in artificially constructed circPSD3 groups. (**G–I**) Relative mRNA levels of *SERPINB2, uPAR*, and *uPA* in 48 paired HCC and non-cancerous tissues. (**J**) Pearson correlation analysis showing a positive correlation between circPSD3 and *SERPINB2* in 48 HCC tissues. (**K**) Immunohistochemical staining revealing the protein levels of SERPINB2 and uPAR in two patients with HCC and PVTT. Scale bar, upper = 100 μm; lower = 20 μm. (**L**) Wound healing assays in the indicated cells. Scale bar = 100 μm. (**M**) Transwell assays showing the migration and invasion of the indicated cells. Scale bar = 100 μm. Data are shown as the mean ± SD. Statistical analyses were performed using unpaired Student’s t-tests (NS, no statistical significance; **p < 0.01; ***p < 0.001; ****p < 0.0001). circPSD3, circRNA pleckstrin and Sect. 7 domain containing 3; HCC, hepatocellular carcinoma; PVTT, portal vein tumor thrombosis; qRT-PCR, quantitative real-time PCR; SERPINB2, serpin family B member 2; uPA, urokinase-type plasminogen activator; uPAR, uPA receptor

To explore the underlying regulatory mechanism of circPSD3 in hepatocellular carcinoma (HCC) metastasis, we conducted RNA-seq analysis on HCC-LM9 cells that were stably transfected with either circPSD3 overexpression or control lentivirus. This approach aimed to identify potential genes and signaling pathways that are influenced by circPSD3 in HCC cells. Based on a differential filtering criteria of (|log2(fold change)|> 1 and p < 0.05), a total of 240 dysregulated genes were identified by RNA-seq, including 42 significantly upregulated and 178 significantly downregulated genes in the circPSD3 overexpression group (Fig. 5A, Additional file 1: Fig. S4A, and Additional file 4: Table S6). Enrichment analysis showed that these aberrantly expressed genes were involved in multiple tumor-related pathways, including the PI3K-AKT, MAPK, focal adhesion, and extracellular matrix (ECM)-receptor interaction pathways (Additional file 1: Fig. S4B, C). qRT-PCR validation of the top five significantly up and downregulated genes was performed in HCC-LM9 and SK-Hep-1 cells. As shown in Fig. 5B, C, the qRT-PCR results were highly consistent with those of RNA-seq. Among these top changed genes, *SERPINB2* was the most upregulated gene after circPSD3 overexpression. SERPINB2 is an endogenous inhibitor of the uPA system. The uPA system plays a pivotal role in the degradation of the ECM and basement membrane during cell invasion. SERPINB2 interacts with uPA to induce endocytosis of uPA–uPAR complexes in a low-density lipoprotein receptor (LDLR)-dependent manner [35, 36]. To further confirm the regulatory role of circPSD3 in the expression of SERPINB2, the expression levels of *SERPINB2* were measured in HCC cells with stable knockdown of circPSD3. The results showed that circPSD3 depletion effectively reduced *SERPINB2* expression in HCC cells (Fig. 5D, E). The regulatory effects of circPSD3 on SERPINB2 and other components of the uPA system (uPA and uPAR) were examined by western blotting (Fig. 5F). These results suggest that SERPINB2 and its regulated uPA system are downstream targets of circPSD3.

To investigate the effect of the uPA system on HCC progression, the mRNA levels of *SERPINB2*, *uPA*, and *uPAR* were measured by qRT-PCR in 48 paired HCC and non-cancerous tissues. *SERPINB2* was significantly downregulated, and *uPA* and *uPAR* were significantly upregulated, in HCC tissues (Fig. 5G–I), consistent with previous studies [37–40]. Pearson correlation analysis showed that *SERPINB2* expression was positively associated with circPSD3 in HCC tissues (Fig. 5J), strengthening the regulatory relationship between circPSD3 and SERPINB2. Immunohistochemical staining of two patients with HCC and PVTT showed that the protein levels of SERPINB2 gradually decreased from non-cancerous to HCC and to PVTT tissues, whereas an inverse trend was observed for uPAR (Fig. 5K), indicating the potential of the uPA system to promote vascular invasion in HCC. In vitro studies showed that SERPINB2 knockdown significantly promoted the migration and invasion of HCC cells (Additional file 1: Fig. S5A–E). Rescue experiments showed that SERPINB2 depletion abrogated the inhibitory effect of circPSD3 on cell migration and invasion (Additional file 1: Fig. S5F, G). Similarly, WX-671, an inhibitor of uPA system, significantly reduced the cell migration and invasion that were induced by circPSD3 knockdown (Fig. 5L, M). Taken together, these results suggest that SERPINB2 and its regulated uPA system mediate the inhibitory effect of circPSD3 on the migration and invasion of HCC cells.

### circPSD3 directly interacts with HDAC1

Next, we investigated the mechanism of circPSD3 regulation of SERPINB2. As circPSD3 is predominantly localised to the cytoplasm of HCC cells, we speculated that circPSD3 may act as a miRNA sponge. However, RIP analysis showed that the AGO2 antibody did not enrich circPSD3, with ciRS-7 serving as a positive control (Additional file 1: Fig. S6). Therefore, we ruled out the possibility of circPSD3 functioning as a miRNA sponge.

Encoding functional peptides is a novel mechanism for some circRNAs, and open reading frame (ORF) and internal IRES are necessary for the translation of circRNAs. The ORF and IRES of circPSD3 were predicted using the circRNADb database (Additional file 1: Fig. S7A), suggesting that circPSD3 has the potential to encode a protein. The dual-luciferase assay showed that the IRES of circPSD3 effectively initiates the translation of luciferase mRNA compared with mutated sequences (Additional file 1: Fig. S7B). We inserted a FLAG coding sequence before the stop codon of the putative ORF of circPSD3 and detected a FLAG-labelled protein in HEK-293T cells (Additional file 1: Fig. S7C), implying that the ORF of circPSD3 may act as a template for translation. To confirm the encoding ability of circPSD3 in the circular isoform, we generated two highly specific antibodies against the 19–35 and 362–488 amino acid sequences of the ORF of circPSD3, which were designed to recognise proteins encoded by circPSD3 (Additional file 1: Fig. S7D). FLAG-tagged ORF and circPSD3 overexpression vectors were successfully transfected into HCC cells (Additional file 1: Fig. S7E). However, western blotting only showed a strong band in HCC cells transfected with the FLAG-tagged ORF vector and not the circPSD3 overexpression vector (Additional file 1: Fig. S7F), suggesting that circPSD3 is a non-coding RNA.

**Fig. 6:**
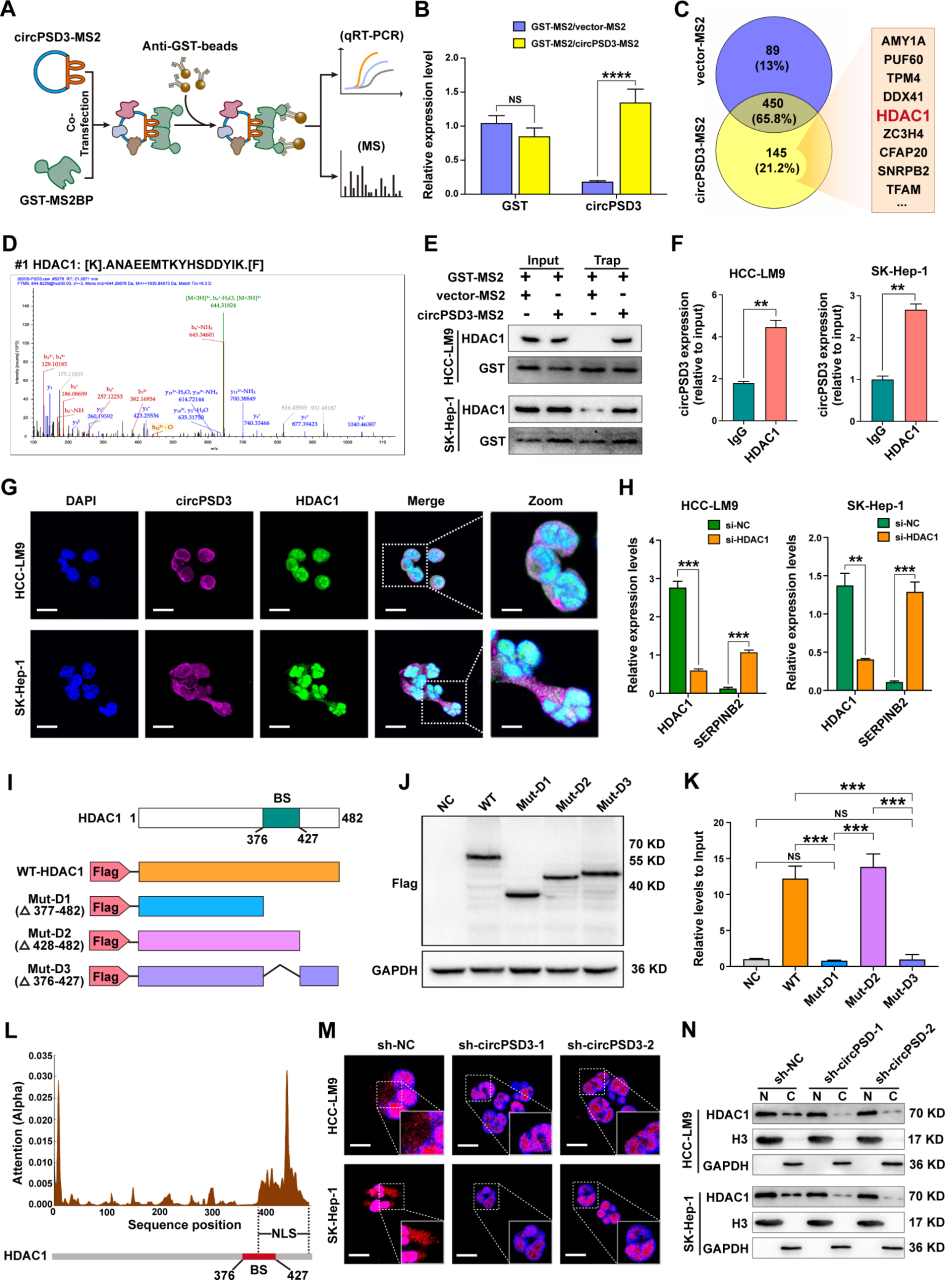
circPSD3 interacts with HDAC1 to inhibit its nuclear translocation. (**A**) Schematic of the TRAP assay. (**B**) Relative expression levels of circPSD3 and GST in HCC-LM9 cells co-transfected with the circPSD3-MS2 and GST-MS2 vectors. (**C**) Venn diagram of circPSD3-interacting proteins, as identified by TRAP assay and LC-MS/MS. (**D**) HDAC1-specific peptide sequence identified using LC-MS/MS. (**E**) TRAP assay and western blotting analysis of the interaction between circPSD3 and HDAC1. (**F**) RIP assay of the enrichment of anti-HDAC1 on circPSD3 in HCC cells. (**G**) FISH and immunofluorescence analysis of the co-localisation of circPSD3 and HDAC1 in the cytoplasm of HCC cells. Scale bar = 20 μm; Scale bar (zoom) = 10 μm. (**H**) Relative expression levels of circPSD3 in HCC cells transfected with HDAC1 siRNA. (**I**) Schematic of the circPSD3 binding site on HDAC1 and FLAG-tagged HDAC1 truncated mutants. (**J**) Western blotting analysis of the protein expression of the negative control, wild-type, and truncated HDAC1 mutants in lysates of HEK-293T cells transfected with the indicated vectors. (**K**) RIP analysis of the enrichment of the negative control, wild-type, and truncated HDAC1 mutants on circPSD3 in HEK-293T cells. (**L**) HDAC1 nuclear localisation signal predicted using DeepLoc-1.0 software (upper) and circPSD3 binding site on HDAC1 (lower). (**M**) Immunofluorescence analysis of the subcellular localisation of HDAC1 in circPSD3 knockdown and control HCC cells. Scale bar = 20 μm. (**N**) Western blotting analysis of HDAC1 expression in the nuclear (N) and cytoplasmic (C) fractions of circPSD3 knockdown and control HCC cells. Data are shown as the mean ± SD. Statistical analyses were performed using unpaired Student’s t-tests (NS, no statistical significance; **p < 0.01; ***p < 0.001). circPSD3, circRNA pleckstrin and Sect. 7 domain containing 3; FISH, fluorescence in situ hybridisation; HCC, hepatocellular carcinoma; HDAC1, histone deacetylase 1; LC-MS/MS, liquid chromatography–tandem mass spectrometry; RIP, RNA immunoprecipitation; TRAP, MS2-tagged RNA affinity purification; siRNA, small interfering RNA

Protein interactions also play an important role in circRNA function. In previous studies, circRNA-interacting proteins were identified by RNA-pulldown assays using a biotin-labelled probe targeting the back-splicing site of circRNA [41, 42]. However, owing to the high GC content (55%) at the back-splicing site, it was difficult to design a probe targeting circPSD3. Instead, we used an MS2-tagged TRAP assay to identify proteins interacting with circPSD3. The circPSD3-MS2 and GST-MS2 vectors were co-transfected into HCC-LM9 cells. Potential circPSD3-binding proteins were detecting using GST antibody-coated magnetic beads (Fig. 6A). qRT-PCR was performed to confirm the upregulation of GST and circPSD3 in HCC cells transfected with these vectors. MS2 sequences were detected by denatured agarose gel electrophoresis and Sanger sequencing (Additional file 1: Fig. S8A, B). Silver staining revealed a specific protein band at ~ 70 KD in the circPSD3-MS2 group (Additional file 1: Fig. S8C). Subsequent LC-MS/MS identified 145 proteins that were uniquely enriched in the circPSD3-MS2 group, 16 of which had more than three unique peptide sequences (Fig. 6C and Additional file 5: Table S7). As expected, LC-MS/MS analysis did not reveal any interaction between circPSD3 and AGO2 (Additional file 1: Fig. S6), consistent with the RIP results.

Given that circPSD3 exerts regulatory control over the expression of SERPINB2, affecting both its mRNA and protein levels. Additionally, RNA-seq results also showed that circPSD3 significantly upregulates the mRNA level of *SERPINB10*, a gene located downstream of SERPINB2 on chromosome 18 (Additional file 1: Fig. S8D). Therefore, we speculated that circPSD3 interacts with a protein that regulates the transcription of SERPINB2. Among circPSD3-MS2-enriched proteins, HDAC1 is a member of the family of histone deacetylases that are involved in transcriptional repression through histone deacetylation (Fig. 6D; Additional file 1: Fig. S8E and F; and Additional file 5: Table S7) [43]. The TRAP assay and western blotting analysis further confirmed the interaction between circPSD3 and HDAC1 (Fig. 6E). The RIP assay also showed that circPSD3 was precipitated by HDAC1 in HCC cells (Fig. 6F). FISH and IF analysis visualised the co-localisation of endogenous circPSD3 and HDAC1 in the cytoplasm of HCC cells (Fig. 6G). Previous studies reported that HDAC1 binds to the promoter of SERPINB2 and regulates the expression of SERPINB2 [44, 45]. Our results also showed that HDAC1 knockdown significantly upregulated the expression of *SERPINB2* in HCC cells (Fig. 6H), with corresponding changes in the protein levels of uPA and uPAR (Additional file 1: Fig. S8G). Hence, we selected HDAC1 as a candidate circPSD3-interacting protein. Subsequently, we sought to determine which region of HDAC1 was responsible for its interaction with circPSD3. According to catRAPID, the 376–427 amino acid sequence of HDAC1 was the most likely site to mediate the interaction between HDAC1 and circPSD3 (Additional file 1: Table S8). To test this hypothesis, wild-type and a panel of truncated HDAC1 mutant plasmids were constructed, each of which was tagged with three copies of FLAG (3 × FLAG) at the NH2 terminus (Fig. 6I). These vectors were successfully expressed in HEK-293T cells (Fig. 6J). RIP assays showed that deletion of the 376–427 amino acid sequence of HDAC1 abolished the interaction between HDAC1 and circPSD3 (Fig. 6K).

### circPSD3 inhibits nuclear translocation of HDAC1

However, western blotting analysis revealed that circPSD3 did not have any impact on the protein levels of HDAC1 (Additional file 1: Fig. S8H). Meanwhile, no modifying proteins were identified by TRAP assay. Using DeepLoc-1.0 software (https://services.healthtech.dtu.dk/services/DeepLoc-1.0/), we found that the nuclear localisation signal of HDAC1 partially overlaps with the putative binding site of circPSD3 (Fig. 6L). Therefore, we speculated that preferential localisation of circPSD3 in the cytoplasm may retain HDAC1 in the cytoplasm, thereby reducing the inhibitory effect of HDAC1 on the transcription of *SERPINB2*. Consistent with this hypothesis, immunofluorescence and nuclear and cytoplasmic fractionation assays showed that circPSD3 knockdown significantly reduced the protein levels of HDAC1 in the cytoplasm of HCC cells (Fig. 6M, N).

Next, wound healing and Transwell assays were performed to determine whether HDAC1 mediates circPSD3 expression during HCC metastasis. HDAC1 depletion significantly suppressed the migration and invasion of HCC cells (Additional file 1: Fig. S9A, B). Co-transfection of HDAC1-targeted siRNA significantly reduced the cell migration and invasion that were enhanced by knocking down circPSD3 (Additional file 1: Fig. S9C, D). These results suggest that HDAC1 mediates the inhibitory effect of circPSD3 on the migration and invasion of HCC cells via interacting with circPSD3.

### circPSD3 provides a novel strategy to inhibit the uPA system

**Fig. 7:**
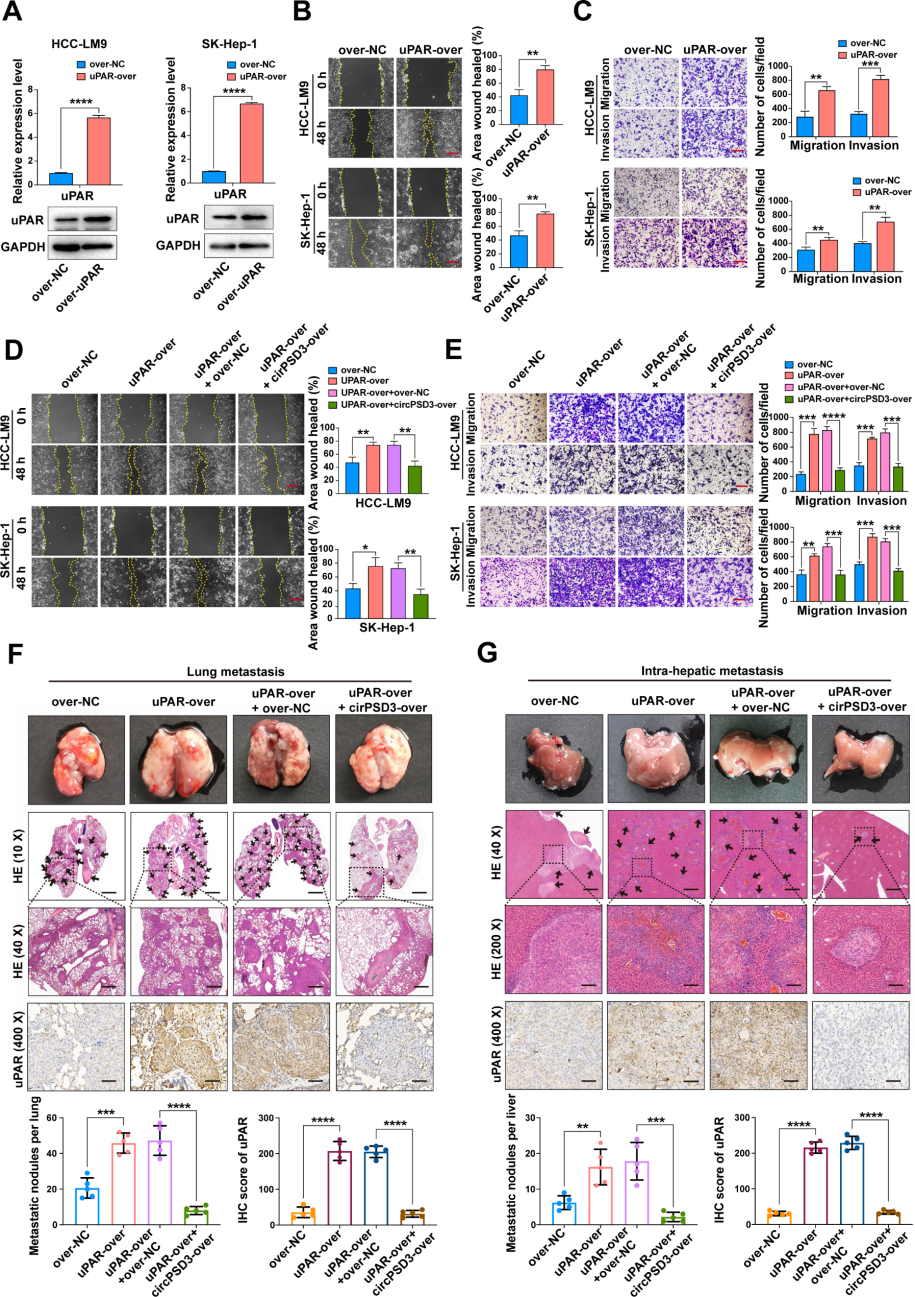
circPSD3 is a novel inhibitor of uPA system. (**A**) uPAR overexpression in HCC cells examined by qRT-PCR and western blotting. (**B**) Representative images and quantification of the wound healing assays in uPAR-overexpressing and control HCC cells. Scale bar = 100 μm. (**C**) Representative images and quantification of the Transwell assays in uPAR-overexpressing and control HCC cells. Scale bar = 100 μm. (**D**) Wound healed assays in the indicated cells. Scale bar = 100 μm. (**E**) Transwell assays showing the migration and invasion of the indicated cells. Scale bar = 100 μm. (**F**) Representative images of H&E and uPAR staining of lung metastatic nodules and quantification of uPAR protein levels in the indicated lung metastasis models. Scale bar (H&E upper) = 1000 μm; Scale bar (H&E lower) = 400 μm; Scale bar (IHC) = 20 μm. (**G**) Representative images of H&E and uPAR staining of intrahepatic metastatic nodules and quantification of uPAR protein levels in the indicated liver orthotopic implantation models. Scale bar (H&E upper) = 400 μm; Scale bar (H&E lower) = 50 μm; Scale bar (IHC) = 20 μm. Data are shown as the mean ± SD. Statistical analyses were performed using unpaired Student’s t-tests (NS, no statistical significance; **p < 0.01; ***p < 0.001; ****p < 0.0001). circPSD3, circRNA pleckstrin and Sect. 7 domain containing 3; HCC, hepatocellular carcinoma; H&E, haematoxylin and eosin; qRT-PCR, quantitative real-time PCR; uPA, urokinase-type plasminogen activator; uPAR, uPA receptor

Given the potency of the uPA system in promoting invasion and metastasis in a wide variety of malignancies, targeting the uPA system is a potential strategy for the treatment of cancer [46]. circRNAs are gradually being recognised as promising treatment agents by virtue of their high stability, low molecular weight, and low immunogenicity [13]. This study has shown that SERPINB2 is a target of circPSD3, suggesting that artificial overexpression of circPSD3 may be a novel approach to inhibit the uPA system. To test this hypothesis, the expression of uPAR was upregulated in HCC cells to mimic an activated uPA system (Fig. 7A). Wound healing and Transwell assays showed that uPAR overexpression significantly increased the migration and invasion of HCC cells (Fig. 7B, C), and this effect was abolished by circPSD3 overexpression (Fig. 7D, E). These results were further strengthened by the pulmonary metastasis and liver orthotopic implantation models, in which co-overexpression of circPSD3 significantly attenuated the lung metastatic lesions and intrahepatic metastatic nodules induced by upregulating uPAR (Fig. 7F, G). However, the inhibitory effect of in vitro synthesised circPSD3 on vascular invasion and metastasis in HCC requires further investigation.

## Discussion

Vascular invasion is a well-known prognostic factor in HCC that is used to guide classification and treatment. Until now, the mechanisms of vascular invasion have remained largely unknown, and treatment options are limited. In this study, using circRNA-seq and qRT-PCR validation, we showed that circPSD3 is downregulated in PVTT tissues. Decreased circPSD3 expression in HCC tissues is an indicator of MVI and is a predictor of poor prognosis in patients undergoing partial hepatectomy. Artificial regulation of circPSD3 affects the migration and invasion of HCC cells, which was demonstrated by a series of in vitro and in vivo experiments. Mechanistic investigations revealed that SERPINB2 is the downstream target of circPSD3. SERPINB2, an endogenous inhibitor of the uPA system, mediates the inhibitory effect of circPSD3 on the invasion and metastasis of HCC cells. To our knowledge, this is the first study to show the relationship between circRNA and vascular invasion, as well as the uPA system.

Theoretically, a single host gene can produce a panel of circRNAs that have different expression patterns and biological functions, due to disparate compositions. The circRNAs produced by PSD3 pre-mRNA have attracted attention in cancer and other diseases. Consistent with our study, a PSD3-derived circRNA was found to be significantly downregulated in ccRCC tissues and was associated with metastasis in patients with ccRCC. circPSD3 overexpression suppressed cell migration and invasion and epithelial–mesenchymal transition in vitro, and inhibited pulmonary metastasis in vivo, in a miR-25-3p/FBXW7-dependent manner [47]. Another PSD3-derived circRNA was found to be significantly upregulated in papillary thyroid cancer tissues and cell lines and was positively associated with a larger tumor size, TNM stage, and lymph node metastasis. circPSD3 knockdown suppressed papillary thyroid cancer cell proliferation and invasion by enhancing the inhibitory effect of miR-7-5p on the expression of METTL7B [48]. The pathological functions of different PSD3-derived circRNAs have also been investigated in hepatic fibrosis [49] and hepatitis C virus (HCV) infection [50]. These researches suggest that PSD3-derived circRNAs play important roles in multiple disease contexts. In-depth investigations of these circRNAs will be meaningful in identifying novel therapeutic targets.

Emerging publications have reported that the biogenesis of circRNAs can be regulated by multiple RBPs. Such as QKI regulates the formation of cSMARCA5 [51], FUS regulates the biogenesis of circCNOT6L [52], and EIF4A3 regulates circMMP9 [53] and circTOLLIP [54] expression. In current study, we demonstrated that TDP43 is an essential regulator on the biogenesis of circPSD3. To our knowledge, this is the first study to reveal the regulating effect of TDP43 on the biogenesis of circRNAs. TDP43 encoded by the *TARDBP* gene mediates multiple aspects of RNA metabolism, including RNA transcription, alternative splicing, and mRNA stabilisation [31]. Previous studies have mainly focused on neurodegenerative diseases, such as Alzheimer’s disease, Parkinson’s disease, and Huntington’s disease [55]. However, studies have also demonstrated a relationship between TDP43 and a variety of malignancies, such as triple-negative breast cancer [56, 57], lung cancer [58], and melanoma [59]. In HCC, several studies have reported that TDP43 is upregulated in HCC tissues. The interaction between TDP43 and *GSK3β* mRNA inhibits the translation of GSK3β, thereby activating the Wnt/β-catenin pathway to enhance HCC cell proliferation and metastasis [33]. TDP43 enhances the stability of ABHD2 mRNA by binding to its 3’UTR. Upregulated ABHD2 enhances lipid metabolism and suppresses apoptosis in HCC cells [34]. TDP43 also inhibits the expression of the miR-520 family by interacting with the miR-520 family promoter. The miR-520/PFKP axis mediates TDP43 regulation of glycolysis in HCC cells [32]. Consistent with these findings, our study showed that TDP43 mRNA levels were significantly higher in HCC tissues than in non-cancerous tissues. This result was strengthened by western blotting in six paired HCC tissues and immunohistochemical staining in a patient with HCC and PVTT. TDP43 expression was inversely correlated with the expression of circPSD3 in HCC tissues, and TDP43 knockdown significantly increased the expression of circPSD3 in HCC cells. Considering the inhibitory effect of circPSD3 on invasion and metastasis, we believe that circPSD3 may be another mediator of TDP43 regulation of vascular invasion and distant metastasis in HCC.

Studies have reported that PSD3-derived circRNAs can sequester miRNAs. However, due to differences in sequences among PSD3-derived circRNAs and the undetectable interaction between circPSD3 and AGO2, sponging miRNAs may not be the primary mechanism by which PSD3-derived circRNAs exert their function. Bioinformatics analysis predicted that circPSD3 may encode novel peptides. The function of the IRES in mediating protein translation and the ORF as a template was also confirmed using appropriate vectors. However, upregulated protein level in circPSD3-overexpressing cells was not detected using specific antibodies against two different amino acid sequences of the predicted encoded peptides. circRNAs tend to form short imperfect intramolecular double-stranded RNAs [60, 61] and interact with diverse molecules [13], these all may largely restrict the recognition of ribosome and the extension of peptide chain. Therefore, the research on translation of circRNAs is still initially, and the encoding notion of circNRAs should be strengthened by more high-quality explorations.

TRAP assay revealed numerous proteins that may potentially interact with circPSD3. Among them, HDAC1, is involved in transcriptional repression through histone deacetylation, allowing histones to wrap DNA tightly. To target specific genomic regions, HDAC1 must interact with DNA binding factors (e.g., transcription factors, nuclear receptors, and DNA methyltransferases). In human corneal and conjunctival epithelial cells, HDAC1 binds to the SERPINB2 promoter, together with pRb2/ p130, E2F5, DNMT1, and SUV39H1 [44]. In MCF-7 breast cancer cells, HDAC1 interacts with CPT1A variant 2 (CPT1AV2). CPT1AV2 knockdown upregulates the levels of HDAC1 and alters the expression of multiple cancer-related genes, including the downregulation of SERPINB2 [45]. HDAC1 is significantly upregulated in HCC tissues compared to matched non-cancerous tissues. Elevated HDAC1 expression in HCC tissues is associated with a higher incidence of portal vein invasion, poorer histological differentiation, and a shorter survival time after hepatectomy [62, 63]. HDAC1 plays important roles in the proliferation, differentiation, apoptosis, and metastasis of HCC cells. In a recent study, HDAC1 interacted with HIF-1α to downregulate the expression of FAM99A, thereby inhibiting HCC metastasis and epithelial–mesenchymal transition by negatively regulating miR-92a during hypoxia [64]. Consequently, we selected HDAC1 as a candidate mediator of the regulatory effect of circPSD3 on SERPINB2 expression and HCC metastasis. The interaction between circPSD3 and HDAC1 was demonstrated in a series of experiments. We found that HDAC1 knockdown enhanced the expression of SERPINB2 in HCC cells. Rescue experiments showed that HDAC1 knockdown attenuated the promoting effects of circPSD3 knockdown on the migration and invasion of HCC-LM9 and Sk-Hep-1 cells. These results suggest that HDAC1 is a key mediator of the inhibitory effect of circPSD3 on HCC metastasis. However, circPSD3 did not have any impact on the protein levels of HDAC1. No protein modifying proteins were identified by LC-MS/MS. HDAC1 is localised to both the nucleus and cytoplasm of HCC cells. The nuclear localising signal of HDAC1 is partially overlaps with the putative binding site of circPSD3. Therefore, we speculated that preferential localisation of circPSD3 in the cytoplasm may retain HDAC1 in the cytoplasm, thereby reducing the transcriptional repression effect of HDAC1. This hypothesis was tested by immunofluorescence and nuclear and cytoplasmic fractionation assays. However, the binding sequence on circPSD3 that mediates its interaction with HDAC1, and the detailed mechanism involved in the cytoplasmic retention of HDAC1, require further investigation.

Degradation of the basement membrane and ECM is primarily mediated by plasmin [65] and is essential for cell migration, invasion, and metastasis [6]. Plasmin is produced by the inactive zymogen, plasminogen [66], which is activated by two types of plasminogen activators: the tissue-type plasminogen activator (tPA) and the uPA [66]. tPA preferentially mediates the degradation of intravascular fibrin deposition [67], whereas uPA, uPAR, and two endogenous inhibitors, plasminogen activator inhibitor-1 (PAI-1; also known as SERPINE1) and PAI-2 (also known as SERPINB2), constitute the uPA system [46]. Once bound to uPAR, uPA catalyses the conversion of plasminogen to plasmin, forming an effective proteolytic enzyme system at the cell surface [68, 69]. Plasmin not only degrades components of the ECM, but also promotes ECM degradation by activating latent MMPs and growth factors [70, 71]. The proteolytic activity of uPA can be suppressed by SERPINE1 or SERPINB2 [72, 73]. After uPA inhibition, uPA–PAI complexes facilitate the interaction between uPAR and low-density lipoprotein receptor, thereby stimulating endocytosis of uPA–PAI complexes and partial recycling of uPAR to the cell surface [74]. Generally, SERPINB2 expression in tumor tissues is negatively associated with cancer growth and metastasis. Using immunohistochemistry, Zhou et al. [38] revealed that the proportion of SERPINB2-positive cells in HCC tissues was significantly lower than that in non-cancerous tissues (26.9% vs. 71.8%, respectively). Negative intratumoral staining of SERPINB2 was independently associated with the presence of PVTT and predicted a poor prognosis. Comparable results were achieved in our study. The expression of SERPINB2 was significantly lower in HCC tissues than in non-cancerous tissues, whereas the reverse was true for the expression of uPA and uPAR. In two patients with HCC and PVTT, the staining intensity of SERPINB2 reduced from normal to HCC and to PVTT tissues. However, the opposite trend was observed for uPAR. These findings further strengthen the ability of the uPA system to regulate HCC cell migration and invasion.

Given the potency of the uPA system in promoting invasion and metastasis in a wide variety of malignancies, targeting the uPA system is a potential strategy for the treatment of cancer [37, 46, 75–77]. In the past, therapeutic agents and approaches have focused on regulating constituents of the uPA system or blocking their biological activity [78–80]. In a previous study, recombinant SERPINB2 and SERPINB2 cDNAs were used to upregulate SERPINB2 expression [72, 81–83]. In this study, we introduced a novel circPSD3 to enhance SERPINB2 expression, thereby inhibiting the uPA system. circRNAs are gradually being recognised as promising treatment agents by virtue of their high stability, low molecular weight, and low immunogenicity [13]. The manageable ectopic expression and in vitro synthesis of stable circRNAs have made circRNA-based therapies possible. For example, a circRNA packaged aptamer exhibited greater suppression of the NF-κB pathway than a linear packaged aptamer [84]. miRNA-122 is indispensable for the HCV life cycle by binding to the 5’UTR of HCV RNA. A synthesised circRNA sponge containing four miRNA-122 binding sites sequestered miRNA-122, thereby inhibiting protein synthesis with greater efficacy than Miravirsen [85]. Heterogeneous nuclear ribonucleoprotein L (hnRNPL), an RNA-binding protein, regulates alternative splicing by binding to short CA repeats of nuclear pre-mRNAs. An artificial circRNA containing 20–100 CA dinucleotides showed high affinity for hnRNPL and sequestered hnRNPL in the cytoplasm, resulted in alternative splicing events similar to those caused by siRNA-mediated hnRNPL depletion [86]. It is noteworthy that the aforementioned circRNA-based treatments have only been studied in cell lines or mouse models. Further researches are needed before these findings can be translated into the clinic.

## Conclusions

**Fig. 8:**
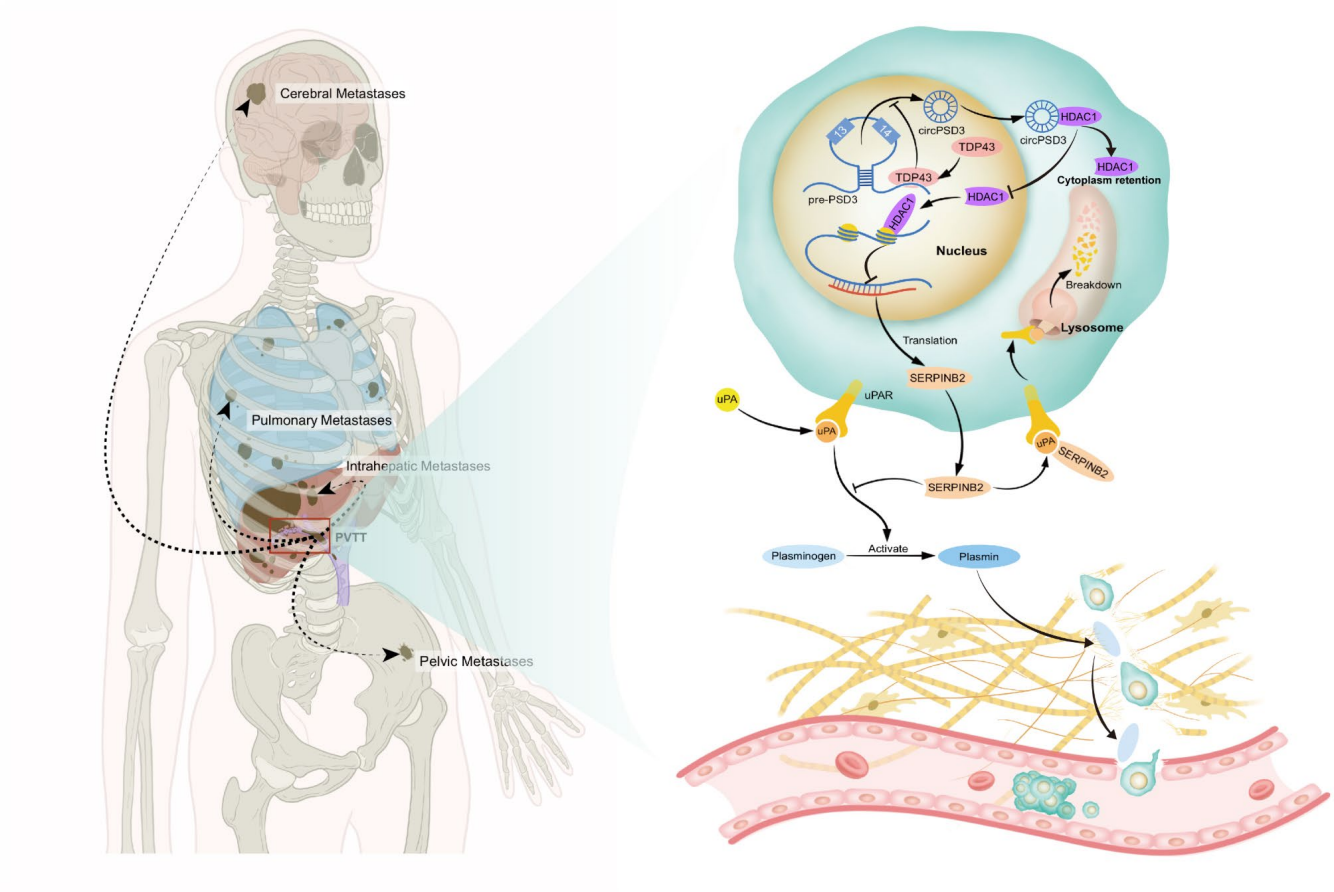
A schematic model visualizing the inhibitory mechanism of circPSD3 in vascular invasion and metastasis of HCC. Vascular invasion is a major route for intrahepatic and distant metastasis (lung, brain, pelvic, etc.) of HCC. circPSD3 is back-spliced by exons 13 and 14 of pre-PSD3. The biogenesis of circPSD3 is negatively regulated by TDP43. Preferential localisation of circPSD3 in the cytoplasm retains HDAC1 in the cytoplasm, thereby reducing the inhibitory effect of HDAC1 on the transcription of SERPINB2. As an endogenous inhibitor of the uPA system, SERPINB2 interacts with uPA to induce endocytosis of uPA–uPAR complexes, thus to inhibit the activation of plasminogen and attenuate the degradation of extracellular matrix and basement membrane by plasmin

circPSD3 is a circRNA that is involved in invasion and metastasis. Decreased circPSD3 in tumor tissues predicted a poor prognosis in patients with HCC after partial hepatectomy. TDP43 contributes to the biogenesis of circPSD3. Mechanistically, circPSD3 interacts with HDAC1, sequestering HDAC1 in the cytoplasm, thereby releasing the transcriptional repression of HDAC1 on SERPINB2. Elevated SERPINB2 inhibits the uPA system and mediates the inhibitory effect of circPSD3 on the regulation of the invasion and metastasis of HCC cells  (Fig. 8). Our findings provide novel insights into treatment strategies targeting the uPA system.

### Electronic supplementary material

Below is the link to the electronic supplementary material.

**Additional file 1**. Fig. S1: Identification of differentially expressed circRNAs in PVTT tissues. Fig. S2: The infection efficiency of HCC-LM9 and SK-Hep-1 cells after treatment with knockdown or overexpression lentiviruses of circPSD3. Fig. S3: circPSD3 has no effect on proliferation of HCC cells. Fig. S4: Identification of down-stream targets of circPSD3. Fig. S5: SERPINB2 mediates the inhibitory effect of circPSD3 on migration and invasion of HCC cells. Fig. S6: RIP assay showed that Anti-AGO2 did not enrich circPSD3. Fig. S7: circPSD3 is a non-coding RNA. Fig. S8: circPSD3 interacts with HDAC1. Fig. S9: HDAC1 mediates the inhibitory effect of circPSD3 on migration and invasion of HCC cells. Table S1. Information of siRNAs used in this study. Table S2. Primers used in this study. Table S5. The relationship between circPSD3 expression in HCC tissues and the clinical characteristics of HCC patients. Table S8. Predicted binding region of HDAC1 to circPSD3

**Additional file 2**. Table S3: The circRNA sequecing data supplied by Novegene

**Additional file 3**. Table S4: The detailed information of circRNAs identified by high-throughput sequencing

**Additional file 4**. Table S6: The differently expressed genes achieved by transcriptome sequencing

**Additional file 5**. Table S7: The proteins identified by MS2-TRAP followed by mass spectrometry

## Data Availability

All data generated or analyzed during this study are included in this published article and its supplementary information files.

## Abbreviations

AGO2: argonaute RISC catalytic component 2
AS: alternative splicing
BM: basement membrane
CCK-8: counting kit-8
circRNA: circular RNA
DMEM: dulbecco’s modified Eagle medium
dsRNA: double-stranded RNA
ECM: extracellular matrix
EMT: epithelial-mesenchymal transition
FBS: fetal bovine serum
FISH: Fluorescence in situ hybridization
GFP: Green fluorescent protein
HBV: hepatitis B virus
HCV: hepatitis C virus
HCC: hepatocellular carcinoma
HDAC1: histone deacetylase 1
HE: hematoxylin and Eosin
hnRNP L: heterogeneous nuclear ribonucleoparticles L
IF: immunofluorescence
IHC: immunohistochemistry
IRES: internal ribosome entry site
LC-MS/MS: Liquid chromatography-tandem mass spectrometry
LDLR: low-density lipoprotein receptor
LncRNAs: long non-coding RNAs
LRP: LDL-related protein
MAPK: mitogen activated kinase-like protein
miRNA: micro RNA
MMP: matrix metalloproteinase
MVI: microvascular invasion
NLS: nuclear localization signal
ORF: open reading frame
PI3K: phosphatidylinositol 3-kinase
PAI-1: plasminogen activator inhibitor-1
PAI-2: plasminogen activator inhibitor-1
PTC: papillary thyroid cancer
PVTT: portal vein tumor thrombosis
qRT-PCR: quantitative real-time PCR
RBP: RNA binding protein
RIP: RNA immunoprecipitation
SERPINB2: serpin family B member 2
SERPINB10: serpin family B member 10
TDP43: TAR DNA binding protein
TNM: tumor-node-metastasis
TRAP: MS2-tagged RNA affinity purification
ULA: ultralow attachment
uPA: urokinase-type plasminogen activator
uPAR: plasminogen activator, urokinase receptor
VLDLR: very-low-density lipoprotein receptor
tPA: tissue-type plasminogen activator
