# Supplementary material for: circPSD3 is a promising inhibitor of uPA system to inhibit vascular invasion and metastasis in hepatocellular carcinoma
Source: Mol Cancer. 2023 Oct 26;22:174. doi: 10.1186/s12943-023-01882-z (PMC10601121; doi:10.1186/s12943-023-01882-z)
Supplement: Supplementary file 1 — Additional file 1. Fig. S1: Identification of differentially expressed circRNAs in PVTT tissues. Fig. S2: The infection efficiency of HCC-LM9 and SK-Hep-1 cells after treatment with knockdown or overexpression lentiviruses of circPSD3. Fig. S3: circPSD3 has no effect on proliferation of HCC cells. Fig. S4: Identification of down-stream targets of circPSD3. Fig. S5: SERPINB2 mediates the inhibitory effect of circPSD3 on migration and invasion of HCC cells. Fig. S6: RIP assay showed that Anti-AGO2 did not enrich circPSD3. Fig. S7: circPSD3 is a non-coding RNA. Fig. S8: circPSD3 interacts with HDAC1. Fig. S9: HDAC1 mediates the inhibitory effect of circPSD3 on migration and invasion of HCC cells. Table S1. Information of siRNAs used in this study. Table S2. Primers used in this study. Table S5. The relationship between circPSD3 expression in HCC tissues and the clinical characteristics of HCC patients. Table S8. Predicted binding region of HDAC1 to circPSD3 [file 12943_2023_1882_MOESM1_ESM.docx]

**Supplementary information includes:**

Supplementary Figure S1–9

Tables S1–S2, S5, S8

Other supplementary material for this manuscript includes:

Tables S3–S4, S6-S7 (Excel format)

**
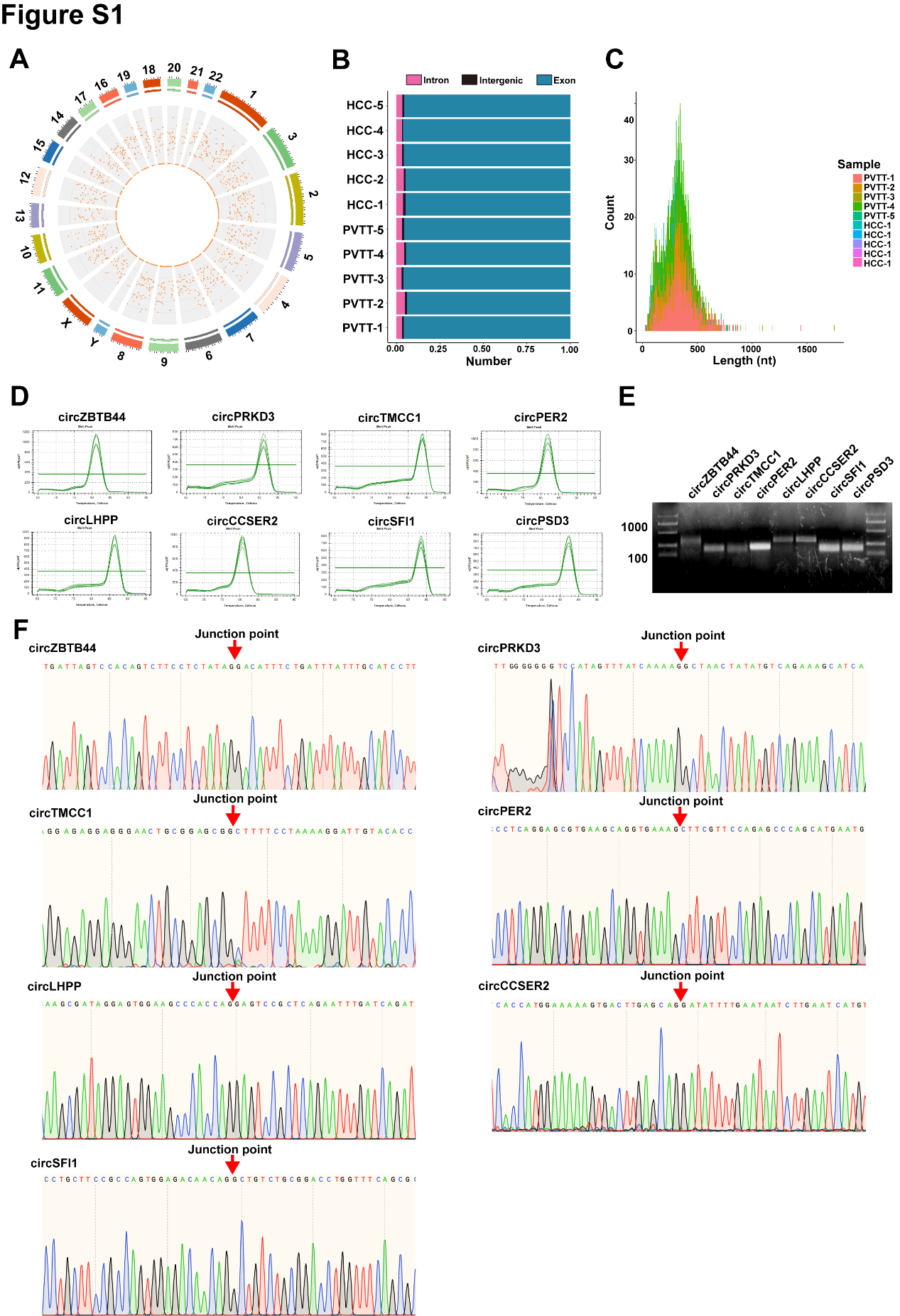
**

**Figure S1. Identification of differentially expressed circRNAs in PVTT tissues.**

**A**. Circos plot indicating the location of differentially expressed circRNAs on human chromosomes. **B.** Classification of identified circRNAs based on genomic origin. **C.** The spliced length range of identified circRNAs. **D.** The melt curves of qRT-PCR product amplified by divergent primers of candidate circRNAs. **E.** The specifity of divergent primers was confirmed by denatured agarose gel electrophoresis. **F.** Sanger sequencing was used to verify the junction point of candidate circRNAs.


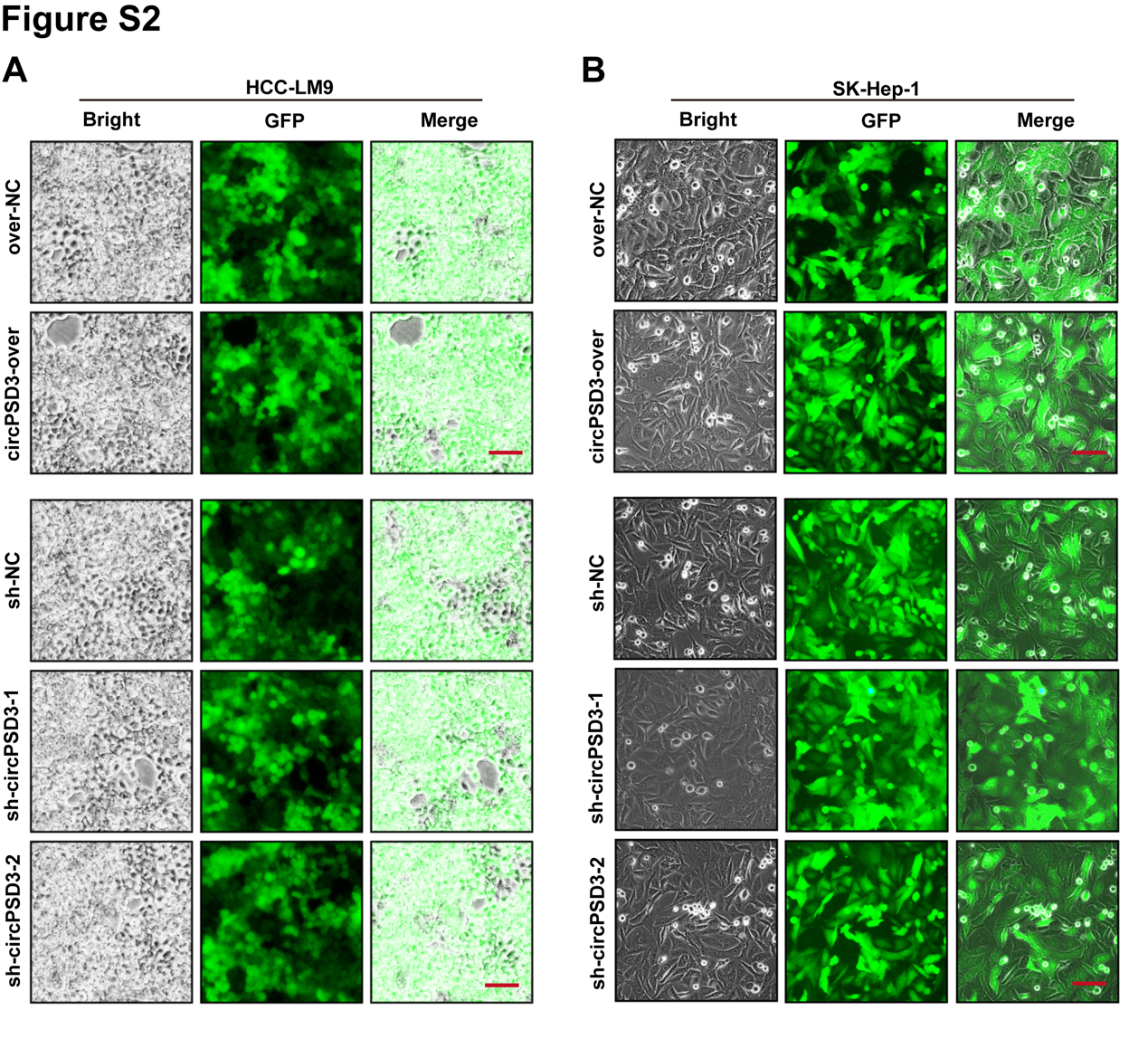


**Figure S2. The infection efficiency of HCC-LM9 and SK-Hep-1 cells after treatment with knockdown or overexpression lentiviruses of circPSD3.** Scale bar = 20 μm.


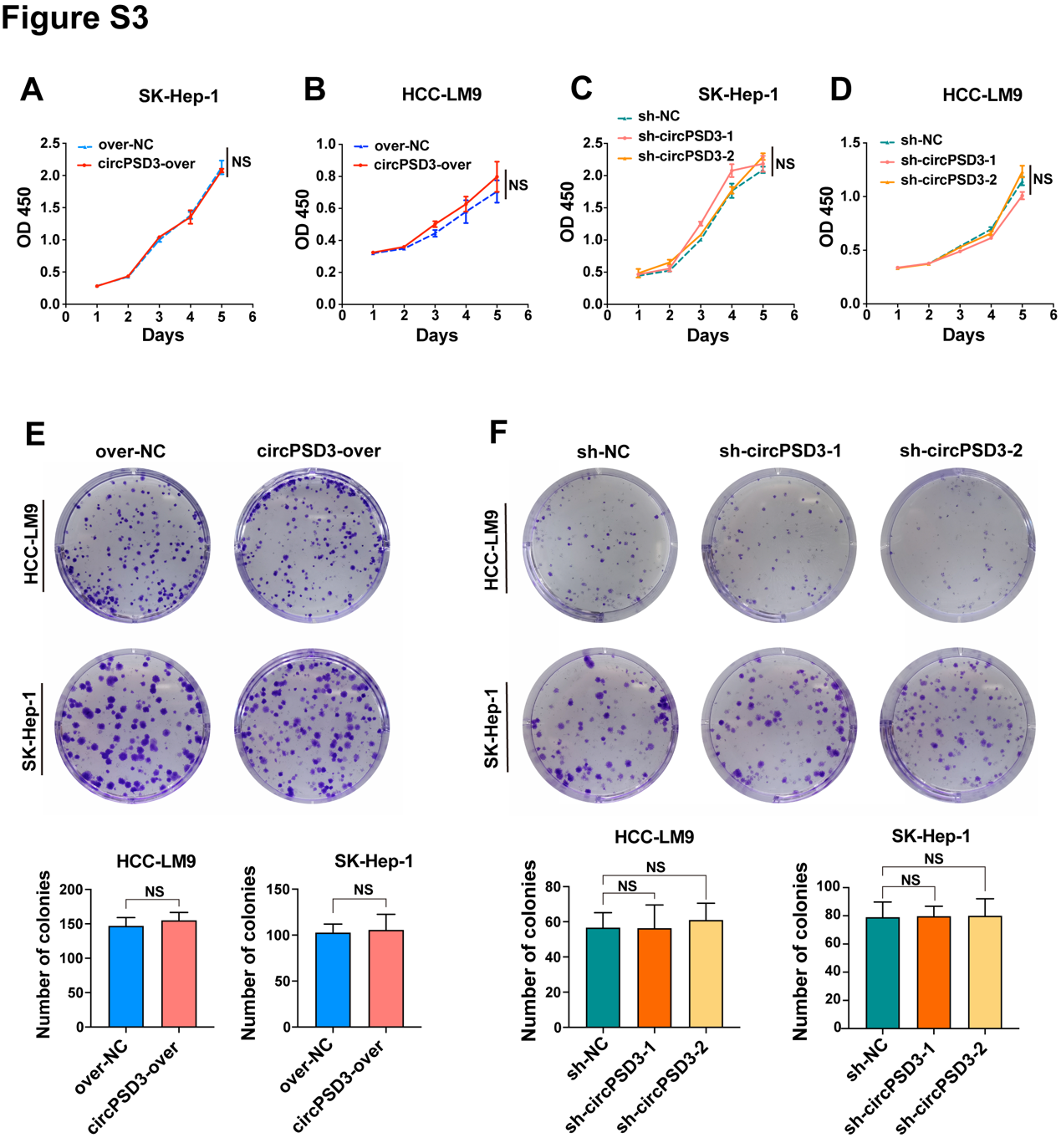


**Figure S3. circPSD3 has no effect on proliferation of HCC cells.**

**A-D.** Cell Counting Kit-8 (CCK-8) assays showed that circPSD3 has no effect on viability of HCC cells. **E** and **F.** Colony formation assay showed that circPSD3 has no effect on the survival of HCC cells.


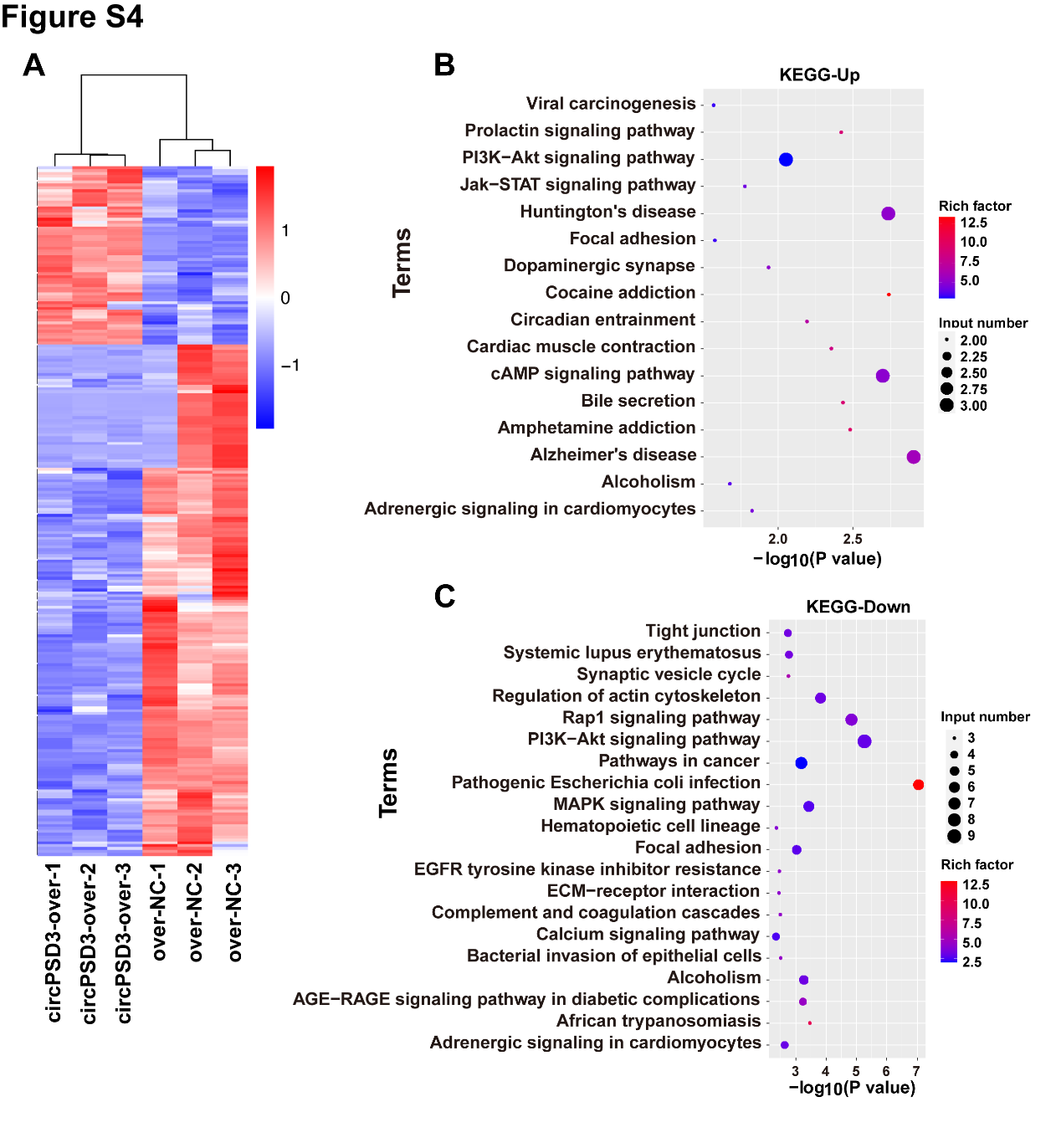


**Figure S4. Identification of down-stream targets of circPSD3.**

**A.** Clustered heat map of the differentially expressed genes between circPSD3-overexpression and control groups. **B.** KEGG analyses of upregulated genes in circPSD3-over group. **C.** KEGG analyses of downregulated genes in circPSD3-over group.


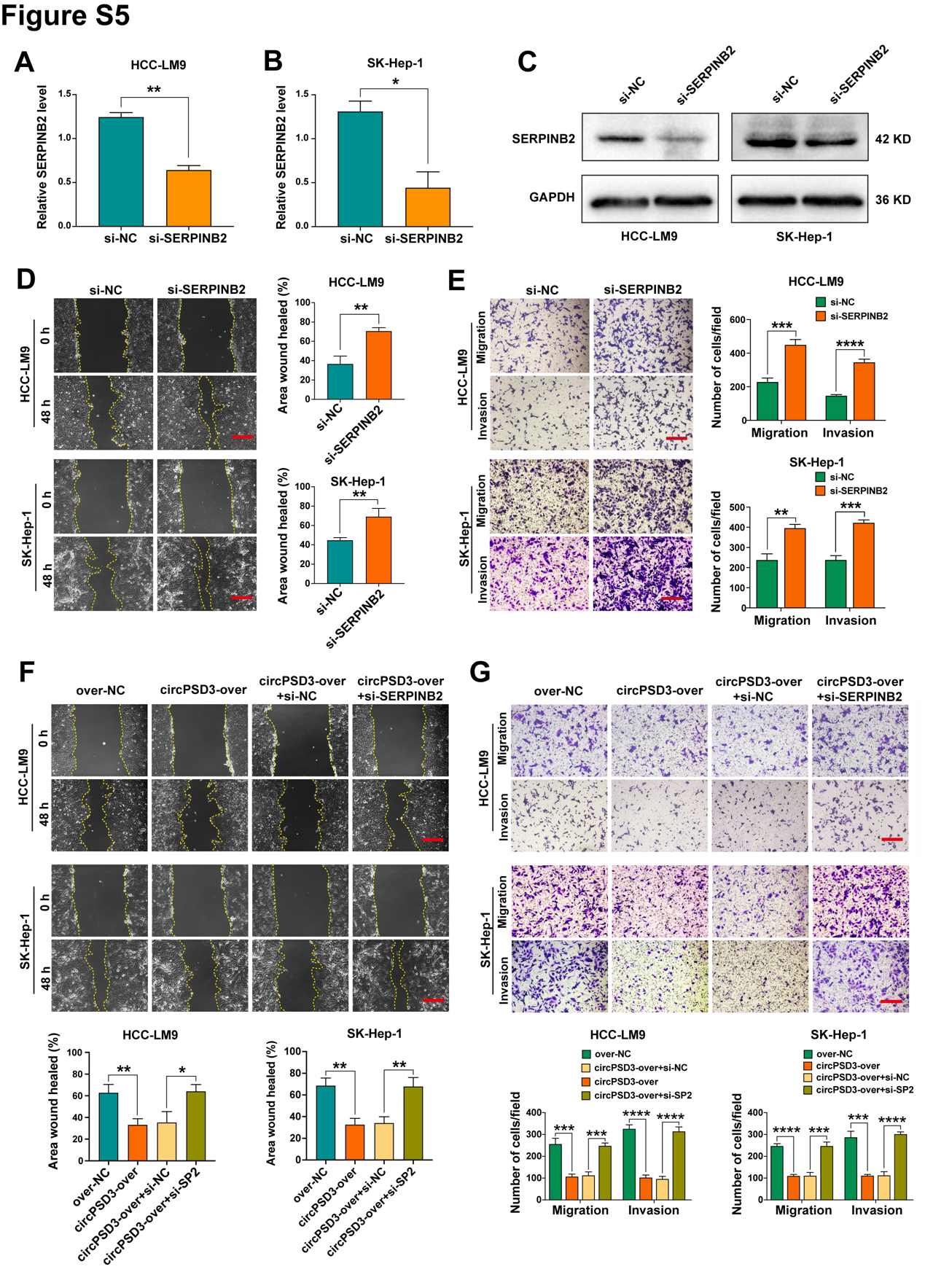


**Figure S5. SERPINB2 mediates the inhibitory effect of circPSD3 on migration and invasion of HCC cells.**

**A-C.** The knockdown of SERPINB2 was examined by both qRT-PCR and Western blot in HCC cells. **D.** Representative images and quantified results of the wound healed assays in SERPINB2-knockdown or control HCC cells. Scale bar = 100 μm. **E.** Representative images and quantified results of the transwell assays in SERPINB2-knockdown or control HCC cells. Scale bar = 100 μm. **F.** Wound healed assays were performed in the indicated cells. Scale bar = 100 μm. **G.** Transwell assays showed the migration and invasion capacity of the indicated cells. Scale bar = 100 μm.


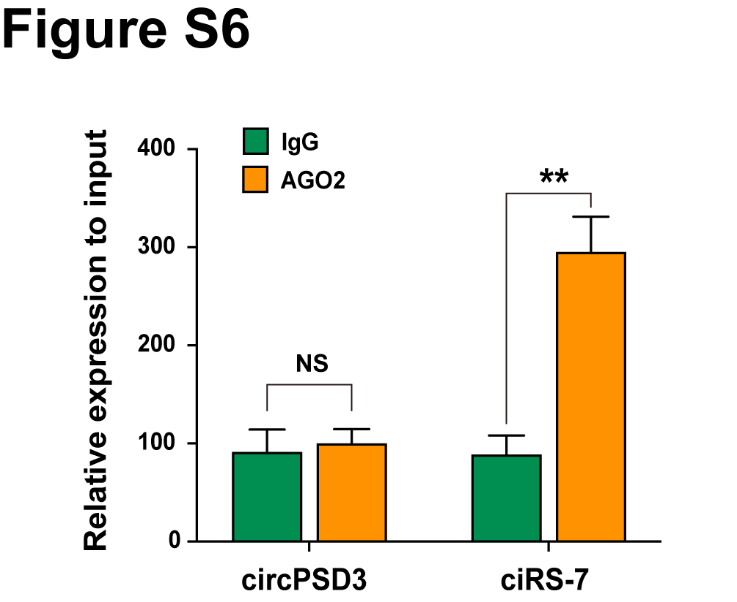


**Figure S6. RIP assay showed that Anti-AGO2 did not enrich circPSD3.** ciRS-7 served as a positive control.


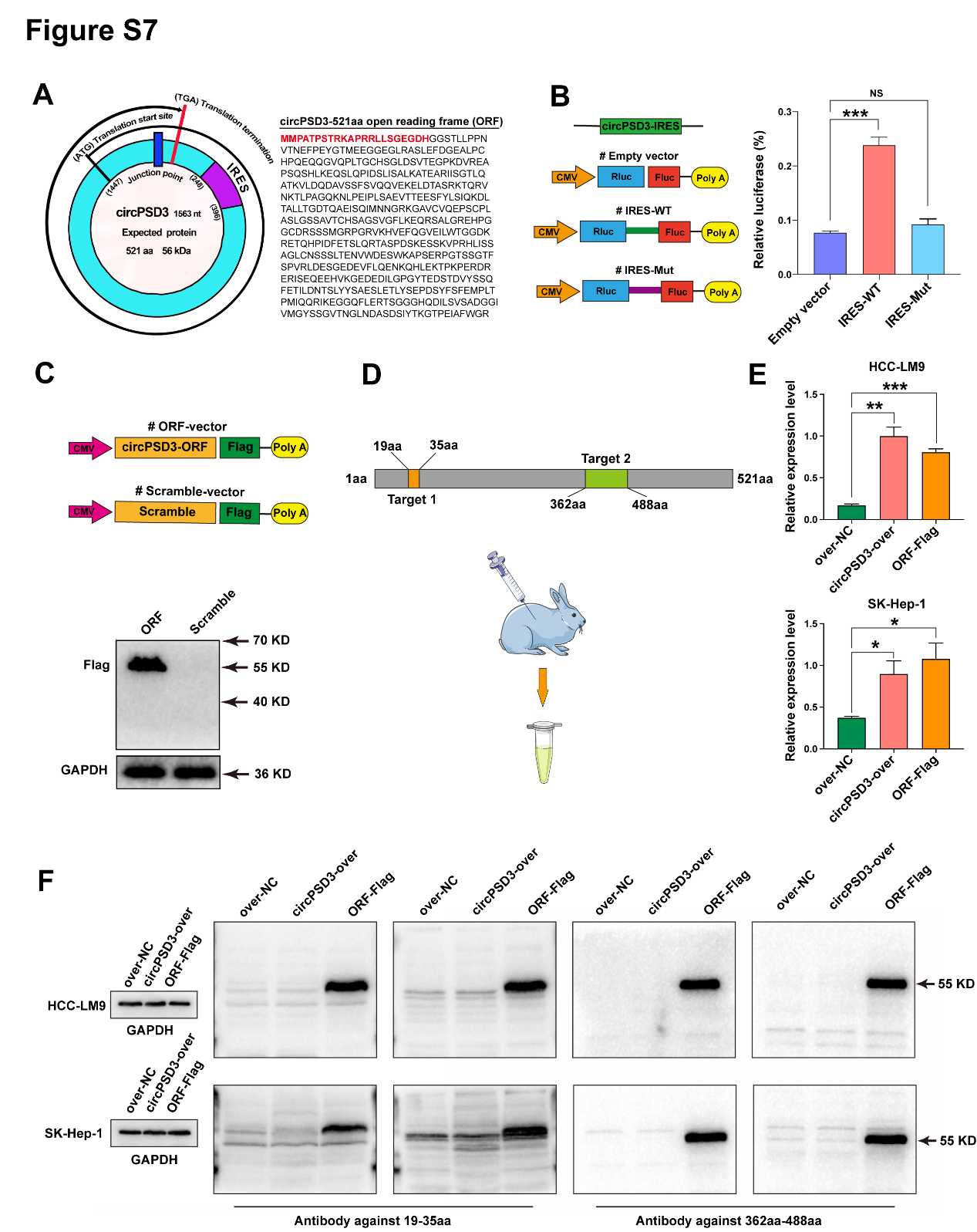


**Figure S7. circPSD3 is a non-coding RNA.**

**A.** Right, schematic illustration showing the potential open reading frame (ORF) and internal ribosome entry site (IRES) of circPSD3 which were predicted by circRNADb database; Left, the possible peptide sequences encoded by circPSD3. **B.** Dual-luciferase assays were employed to evaluate the ability of the IRES of circPSD3 in initiating the protein translation. **C.** Flag-tagged vector was constructed to confirm whether the ORF of circPSD3 can serve as a translation template. **D.** Schematic illustration showing the specific region used to design antibodies of proteins encoded by circPSD3. **E.** qRT-PCR analysis showed that circPSD3-overexpression and Flag-ORF vectors were successfully transfected into HCC cells. **F.** Western blot assays showed that that a strong band only displayed in HCC cells transfected with Flag-ORF vector other than circPSD3-overexpression vector, suggesting that circPSD3 is a non-coding RNA.


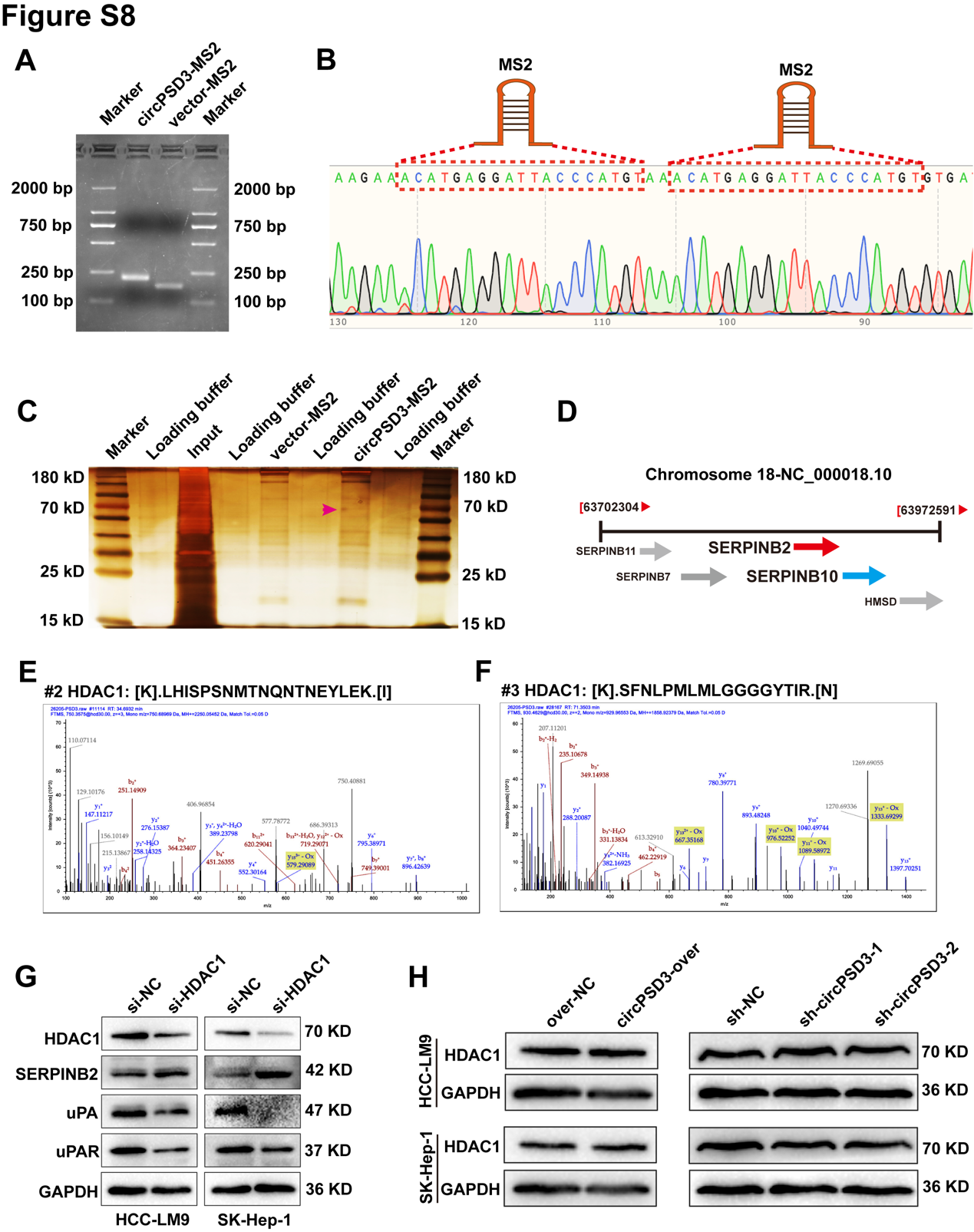


# Figure S8. circPSD3 interacts with HDAC1.

# A. Denatured agarose gel electrophoresis of qRT-PCR products from HCC cells after transfection with circPSD3-MS2 or control vector. B. The 2×MS2 sequences were verified by Sanger sequencing. C. Silver staining of the TRAP elute from HCC-LM9 cells. D. Schematic illustration displaying the chromosome location of SERPINB2 and SERPINB10. E and F. Another two specific peptide sequences of HDAC1 were acquired using MS. G. Western blot assay was performed to detect the expression of uPA system proteins in circPSD3-knockdown and negative control HCC cells. H. Western blot assay showed that circPSD3 has no effect on the protein levels of HDAC1 in HCC cells.


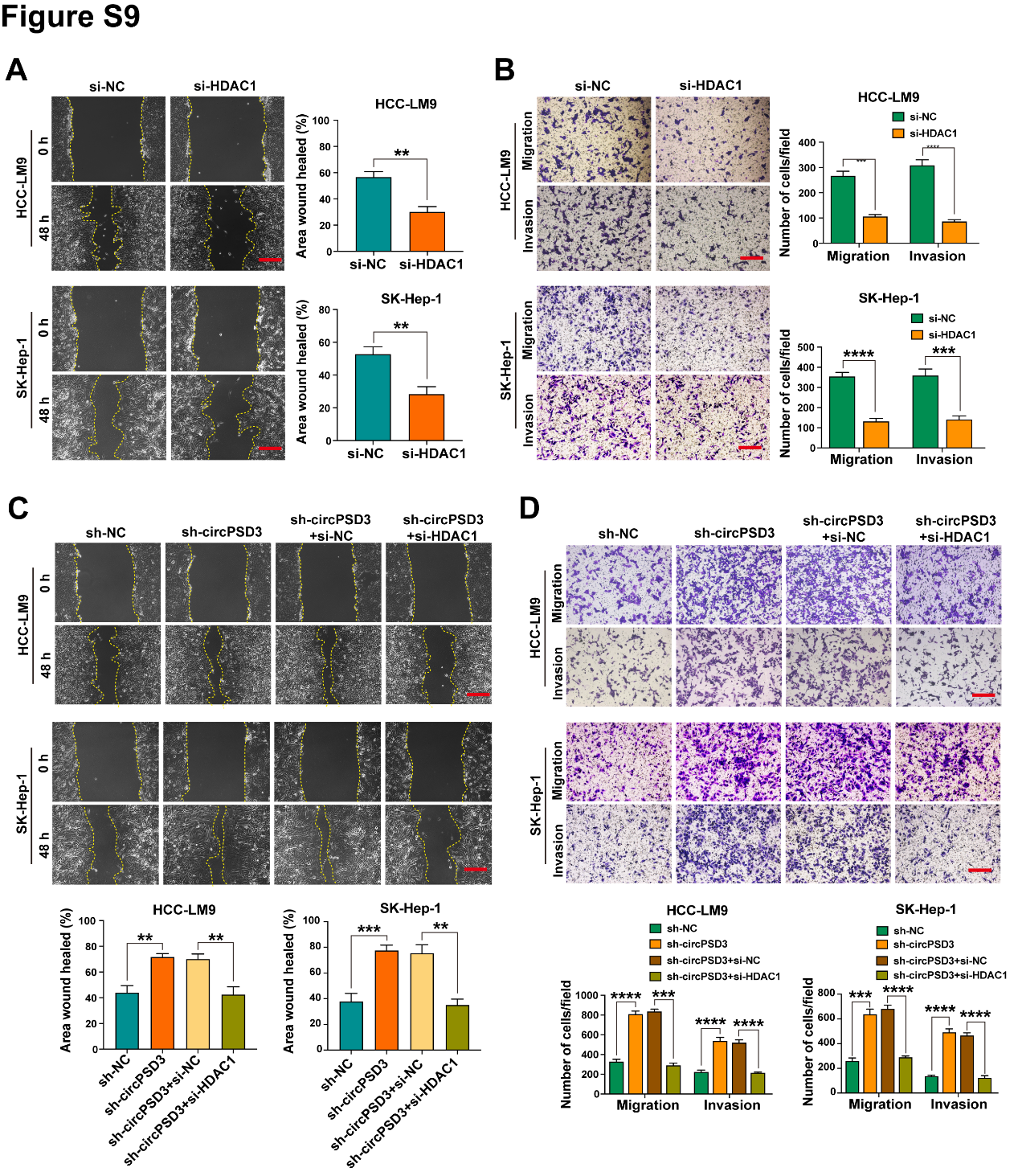


**Figure S9. HDAC1 mediates the inhibitory effect of circPSD3 on migration and invasion of HCC cells. A.** Representative images and quantified results of the wound healed assays in HDAC1-knockdown or control HCC cells. Scale bar = 100 μm. **B.** Representative images and quantified results of the transwell assays in HDAC1-knockdown or control HCC cells. Scale bar = 100 μm. **C.** Wound healed assays were performed in the indicated cells. Scale bar = 100 μm. **D.** Transwell assays showed the migration and invasion capacity of the indicated cells. Scale bar = 100 μm.

**Table S1. Information of siRNAs used in this study**

| **Name** | **Target sequence** | **Supplier** |
| --- | --- | --- |
| si-circPSD3-1 | GGAAGGTGATCATGGAGGA | Ribobio |
| si-circPSD3-2 | GGGAAGGTGATCATGGAGG | Ribobio |
| si-TDP43-1 | CAATAGCAATAGACAGTTA | Ribobio |
| si-TDP43-2 | GCTTCGCTACAGGAATCCA | Ribobio |
| si-SERPINB2 | GCAGTAGACTTCCTAGAAT | Ribobio |
| si-HDAC1 | GAGTCAAAACAGAGGATGA | Ribobio |
| si-NC | CCTAAGGTTAAGTCGCCCTCG | Ribobio |

**Table S2. Primers used in this study**

| **Name** | **Forward primer** | **Reverse primer** |
| --- | --- | --- |
| circZBTB44 | GAAGTGAACAAGTCCAAGAGG | GTCCATCATTTCGCAGCATA |
| circPRKD3 | GCTATTCCTGCTGTGCTTCC | AGATGAATGGGTCCATCGAG |
| circTMCC1 | AGGGAAGTCCCCAAATCAAC | GGCATTTGCTTCAACAGTGA |
| circPER2 | ACCTCCCTGCAGACAAGAAG | TTTCGTTGGTCTCATGTCCA |
| circLHPP | GGGAAGCCTTCTCCTGAGTT | CACACAGTTTGGGTTGGATG |
| circCCSER2 | GCCACCCTGACCACTATCAT | CGGTCAAAGCGGTTCATATT |
| circSFI1 | ACTGCTGGCACAGAGACTCA | GTCTCTGCTGGGCTGACCT |
| circPSD3 | CAGCGACTCCATCTACACGA | GCCTCACCATCAAATTCCAGA |
| mPSD3 | GGATCTCAGACTGCCGTAGC | GCTATGGGCCAGACTCTCAG |
| ciRS-7 | TCAACTGGCTCAATATCCATGTC | ACCTTGACACAGGTGCCAT |
| TDP43 | CCTTGCGTTCATAGCGTTGATAC | TGCCATAGGAATACTGTCTACATGC |
| A (Figure 2B) | CCCCTTTTTGCATATGAAGC | AAAGACAGGGCCCAGTAGGTA |
| B (Figure 2B) | TTGGGATGAATCCTGGAAAG | GTACACGTCGGTGGAGTCCT |
| C (Figure 2B) | CCTGACGTGTTGAAGGAAGG | CAAATAACAGGCATGGTGGA |
| D (Figure 2B) | TCCACCATGCCTGTTATTTG | AACCTGAGGATGACGAGCAT |
| E (Figure 2B) | TCCTCAGGTTATCTACCTGTGAC | GGGCAGACTGCTCAAAAAGA |
| SERPINB2 | CAGCACCGAAGACCAGATGG | CCTGCAAAATCGCATCAGGATAA |
| SERPINB10 | GCCAAAGCAAAGCTGATTTC | GGGGGAGCATAATCTTCCAT |
| CD24 | GCCAGTCTCTTCGTGGTCTC | CCTGTTTTTCCTTGCCACAT |
| APOE | GGTCGCTTTTGGGATTACCT | TCCAGTTCCGATTTGTAGGC |
| LLGL2 | CGGGACCTGTTCCAGTTTAAC | CGTCACAGCGTTGTTCTCC |
| RAB20 | CTATGATGTGAATCACCGGCAG | GGTCCCCAGCGTCCATATTG |
| TNC | TCCCAGTGTTCGGTGGATCT | TTGATGCGATGTGTGAAGACA |
| MPP4 | CAGCAGATGGTGTACGTCCG | CTGGTACGGCTGAGACCAC |
| GLIS3 | AGAATGGCCTTGATCTAGGGG | GTGCCAAAAAGGTAGGATGGTAA |
| ROBO4 | CAGCGCAGGACATAGGGAG | GGCCGTGTAAGATTGGGCA |
| uPA | CTGTCACCTACGTGTGTGGAG | TGAGCGACCCAGGTAGACG |
| uPAR | TGTAAGACCAACGGGGATTGC | AGCCAGTCCGATAGCTCAGG |
| GST | TTTGTATGAGCGCGATGAAG | ACGCTCTTTTGGACAACCAC |
| HDAC1 | CGCCCTCACAAAGCCAATG | CTGCTTGCTGTACTCCGACA |
| β-actin | GTGGCCGAGGACTTTGATTG | CCTGTAACAACGCATCTCATATT |
| U6 | CTCGCTTCGGCAGCACA | AACGCTTCACGAATTTGCG |
| GAPDH | GGAGCGAGATCCCTCCAAAAT | GGCTGTTGTCATACTTCTCATGG |
| Divergent-GAPDH | GGCCTCCAAGGAGTAAGA | GCCCAATACGACCAAATCA |

**Table S5. The relationship between circPSD3 expression in HCC tissues and the clinical characteristics of HCC patients**

| Clinical parameters | circPSD3-low (n=61) | circPSD3-high (n=79) | P value |
| --- | --- | --- | --- |
| Age (years) |  |  | 0.95 |
| ≥ 60 | 19 | 25 |  |
| < 60 | 42 | 54 |  |
| Gender |  |  | 0.16 |
| Male | 56 | 65 |  |
| Female | 5 | 14 |  |
| HBV-DNA (copies/ml) |  |  | 0.914 |
| < 10^3^ | 29 | 40 |  |
| ≥ 10^3^ | 32 | 39 |  |
| AFP (ng/ul) |  |  | 0.012* |
| < 400 | 28 | 53 |  |
| ≥ 400 | 33 | 26 |  |
| Tumor size (cm) |  |  | 0.073 |
| < 5 | 19 | 36 |  |
| ≥ 5 | 42 | 43 |  |
| Tumor number |  |  | 0.04* |
| Single | 38 | 61 |  |
| Multi | 23 | 18 |  |
| Differentiation |  |  | 0.133 |
| I+II | 28 | 47 |  |
| III+IV | 33 | 32 |  |
| MVI |  |  | <0.001* |
| Absent | 25 | 66 |  |
| Present | 36 | 13 |  |
| Tumor capsule |  |  | 0.577 |
| complete | 28 | 31 |  |
| Infiltrate | 33 | 46 |  |
| Liver cirrhosis |  |  | 0.482 |
| No | 37 | 41 |  |
| Yes | 24 | 38 |  |

HBV-DNA, hepatitis B virus deoxyribonucleic acid; AFP, α fetal protein; MVI, microvascular invasion.

^*^Statistical significance.

**Table S8. Predicted binding region of HDAC1 to circPSD3**

| **#** | **HDAC1  Protein region** | **circPSD3 RNA region** | **Interaction Propensity** | **Discriminative  Power** | **Normalized  Score** |
| --- | --- | --- | --- | --- | --- |
| 1 | 376-427 | 694-755 | 17.55 | 47 | 3.83 |
| 2 | 376-427 | 691-752 | 16.99 | 45 | 3.71 |
| 3 | 376-427 | 574-635 | 15.02 | 42 | 3.3 |
| 4 | 376-427 | 1381-1442 | 14.9 | 40 | 3.27 |
| 5 | 376-427 | 661-722 | 13.74 | 37 | 3.03 |
| 6 | 407-458 | 694-755 | 13.43 | 37 | 2.96 |
| 7 | 232-283 | 694-755 | 13.12 | 37 | 2.9 |
| 8 | 407-458 | 691-752 | 13.1 | 37 | 2.89 |
| 9 | 376-427 | 571-632 | 13.02 | 37 | 2.88 |
| 10 | 232-283 | 691-752 | 12.67 | 35 | 2.8 |
| 11 | 401-452 | 694-755 | 12.63 | 35 | 2.8 |
| 12 | 276-327 | 694-755 | 12.57 | 35 | 2.78 |
| 13 | 401-452 | 691-752 | 12.31 | 35 | 2.73 |
| 14 | 376-427 | 664-725 | 12.24 | 35 | 2.71 |
| 15 | 376-427 | 604-665 | 12.22 | 35 | 2.71 |
| 16 | 276-327 | 691-752 | 12.17 | 35 | 2.7 |
| 17 | 382-433 | 694-755 | 11.76 | 33 | 2.61 |
| 18 | 376-427 | 1384-1445 | 11.74 | 33 | 2.61 |
| 19 | 376-427 | 601-662 | 11.48 | 33 | 2.55 |
| 20 | 407-458 | 661-722 | 11.45 | 33 | 2.55 |
